# 1-Amino-but-3-enes scavenge formaldehyde and glyoxylic acid

**DOI:** 10.1038/s42004-025-01873-9

**Published:** 2026-01-12

**Authors:** Natasha F. A. Bulman, Vicki L. Emms, Liam A. Thomas, Lilla Beja, Richard J. Hopkinson

**Affiliations:** Leicester Institute of Structural and Chemical Biology and School of Chemistry, University of Leicester, Henry Wellcome Building, Lancaster Road, Leicester, LE1 7RH UK

**Keywords:** Chemical tools, Chemical modification, Solution-state NMR

## Abstract

Reactive carbonyl compounds are common pollutants and endogenous metabolites that are often toxic at high concentrations. Removal/detoxification of carbonyl compounds requires selective small molecule scavengers; however, few molecules suitable for this task have been fully characterised. Here, we report NMR-based kinetic and selectivity studies on representative 1-amino-but-3-enes, which are reported to be selective formaldehyde scavengers. Our experiments reveal that 1-amino-but-3-enes containing phenyl groups at position 1 react with formaldehyde via a 2-aza-Cope rearrangement. However, they also react with other carbonyl compounds, including the biologically relevant 1,2-dicarbonyl compound glyoxylic acid. The most efficient and promiscuous scavenging compound promoted the growth of *Escherichia coli* cells, while studies on cell lysate revealed potential for aldehyde sequestration. Overall, our analyses suggest that 1-amino-but-3-enes can be used to scavenge a variety of toxic carbonyl compounds and may be used in imaging and quantification studies, as well as for biomedical applications.

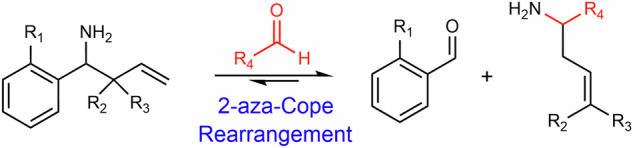

## Abstract (web summary)

Reactive carbonyl compounds pose toxicity challenges as pollutants and metabolites; however, synthetic scavengers for detoxification remain underdeveloped. Here, the authors demonstrate that 1-amino-but-3-enes, particularly those with phenyl groups, selectively scavenge formaldehyde and glyoxylic acid, offering potential for biomedical applications.

## Introduction

Organic small molecules containing aldehyde or ketone functional groups (reactive carbonyls, RCs) are common environmental pollutants and metabolites in humans and other organisms^1^. Above threshold levels, RCs are usually toxic, although many RCs are proposed to have additional ‘healthy’ functions, e.g. in metabolism and signalling. These effects are likely due to reactions between RCs and other biomolecules^2–4^.

Formaldehyde (HCHO), the simplest RC, is produced in vivo during enzyme-catalysed demethylation reactions (e.g. by histone and DNA demethylases) and reacts to form hydroxymethylated and cross-linked adducts with proteins and nucleic acids^5–10^. These adducts are proposed to induce toxicity and carcinogenicity in humans^11–13^, although there are also reported healthy roles in sensing and one-carbon metabolism^14–17^. Similar adducts are reported to form between biomolecules and 1,2-dicarbonyl compounds such as glyoxal and glyoxylic acid (GA), which are produced in human cells during sugar metabolism^18–20^. 1,2-Dicarbonyl-derived adducts include glycation end products, which accumulate on proteins and lipids and are biomarkers for degenerative diseases such as diabetes and Alzheimer’s disease^20,21^. GA is also an important intermediate in the glyoxylate cycle, a metabolic pathway absent in animals that enables adenosine triphosphate biosynthesis from acetyl-coenzyme A^4^. The GA shunt is essential for bacterial growth on fatty acids and acetate, and is therefore a therapeutic target against many bacteria.

Intracellular RC concentrations are regulated by a number of metabolic pathways. While modulation of enzymes involved in these pathways, particularly RC biosynthesis enzymes, by small molecules might be useful for preventing RC overload and/or inhibiting the glyoxylate cycle, such approaches are often limited by off-target effects. An alternative strategy for reducing RC concentrations is to employ cell-penetrant, small-molecule RC scavengers that react with RCs to form harmless stable products that can potentially be analysed for RC imaging and/or quantification studies^22–31^. However, for biologically relevant RC scavenging to be successful, scavengers must react efficiently, and importantly, selectively, with RCs. Identifying suitable compounds is extremely challenging due to RCs’ promiscuous and often dynamic reactivity.

One reported scavenger class, substituted 1-amino-but-3-enes, reacts with HCHO via a 2-aza-Cope rearrangement and has been used in HCHO imaging studies when coupled to a fluorophore or chemiluminescent group^32–35^. However, while these seminal studies conducted selectivity analyses with some RCs and demonstrated faster reactions with methylated 1-amino-but-3-enes (presumably via a Thorpe-Ingold effect), the full reaction kinetics and selectivity profiles of the 1-amino-but-3-ene scaffold have not been fully determined, which hinders its use in quantification and biomedical applications. These studies also did not conduct full NMR analyses on the reactions, thus precluding observation of transient intermediates. We therefore report NMR-based analyses on the reactions of representative 1-amino-but-3-enes with RCs. Our results reveal that 1-amino-1-phenyl-but-3-enes preferentially scavenge HCHO over enolisable carbonyls but react efficiently with GA under physiologically relevant conditions. Time-course studies indicate that the reaction efficiencies of these 1-amino-but-3-enes with HCHO and GA are dependent on both the scavenger substitution pattern and the pH of the reaction mixture, which suggests these factors can be modified to invoke selectivity. Importantly, a 1-amino-but-3-ene containing an ortho-phenolic group and two methyl groups at the allylic carbon was observed to react preferentially with GA over HCHO at pH 7.4, while scavenging of HCHO and GA was detectable in bacterial cell lysate.

Overall, our results indicate that the 1-amino-but-3-ene scaffold reacts efficiently with a subset of biologically relevant RCs, and therefore has clear potential in cellular imaging, quantification and biomedical studies. However, care must be taken to ensure appropriate selectivity, particularly in quantitative assays.

## Results and discussion

**Scheme 1:**
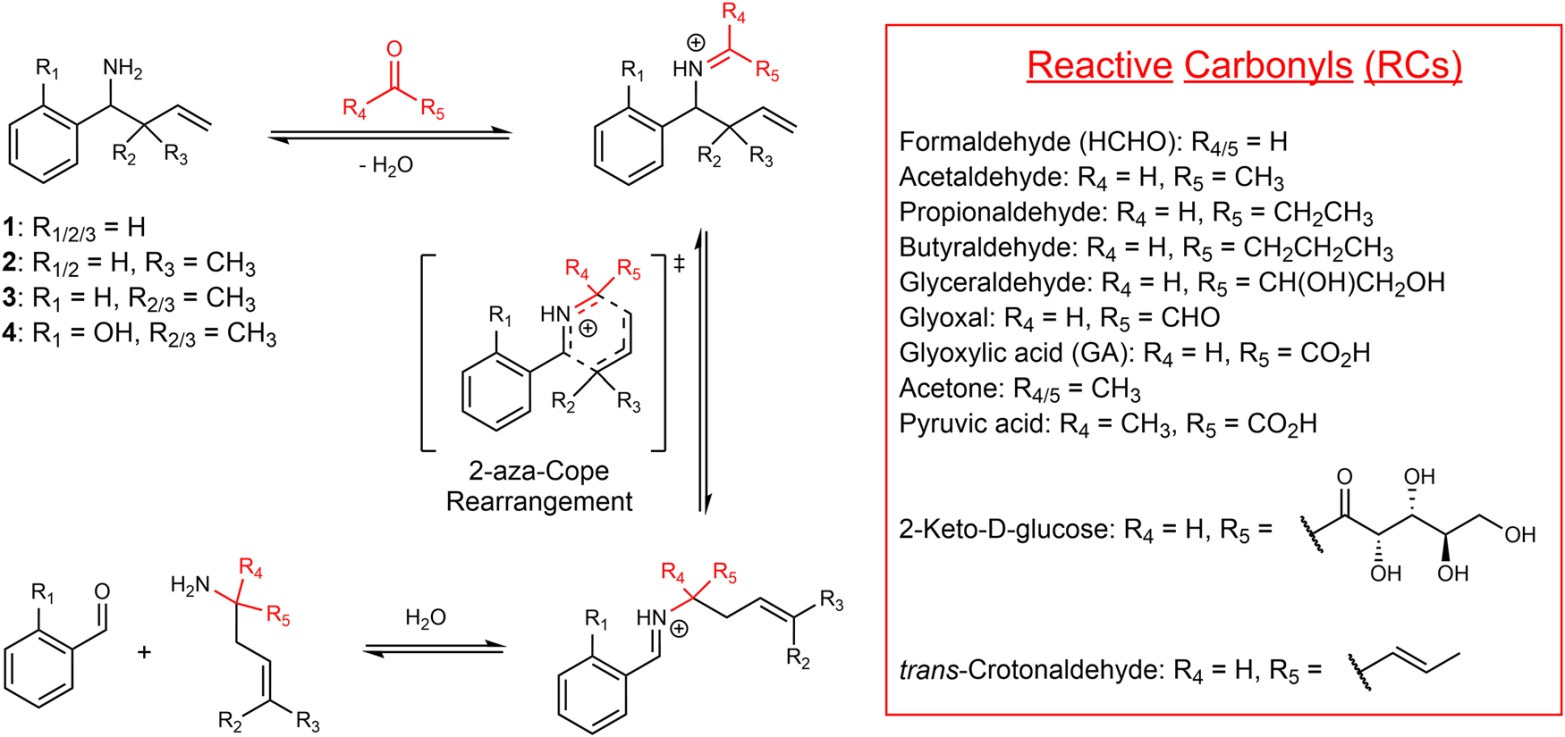
1-Amino-but-3-enes and reactive carbonyls (RCs) react via a 2-aza-Cope rearrangement. (Left) Reaction scheme showing reactions between representative 1-amino-but-3-enes **1**–**4** and RCs. The structures of scavengers **1**–**4** are shown in the top left. (Right) Structures of RCs used in this study.

Initially, we designed and synthesised three representative 1-amino-but-3-enes for use in NMR-based selectivity and kinetic experiments (Scheme 1). These compounds each contain a phenyl group and a benzylic primary amine but differ in their substitution pattern at the allylic carbon. Di-methylation at this position is reported to increase the rate of reaction of 1-amino-but-3-enes with HCHO relative to the unmethylated analogue, although the effect of mono-methylation has not been determined^32^. We were therefore keen to test unmethylated, mono-methylated and di-methylated variants (**1**–**3**, Scheme 1). These compounds were synthesised from benzaldehyde and appropriately substituted allyl boronic pinacol esters.

**Fig. 1:**
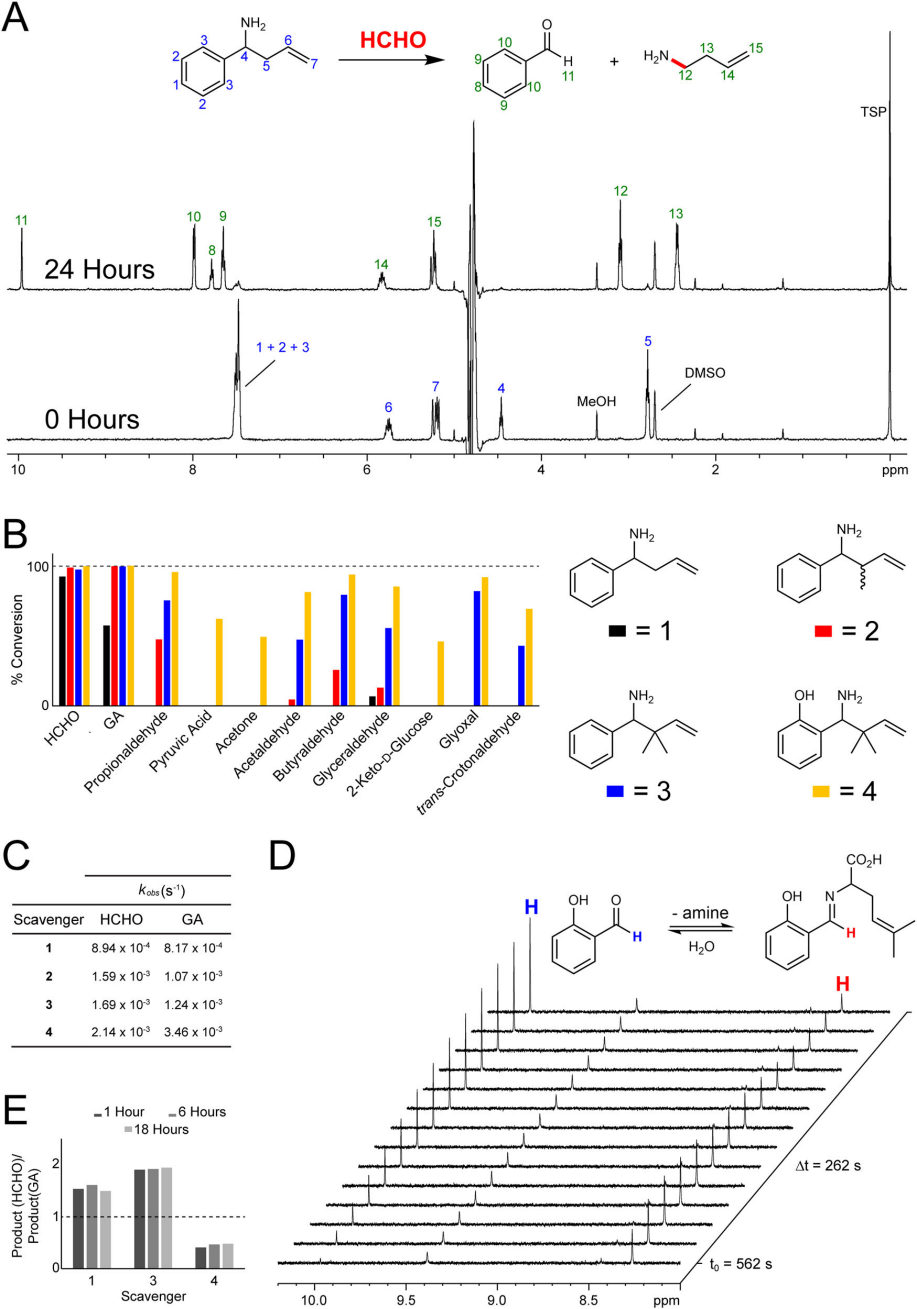
Phenyl group-containing 1-amino-but-3-enes react with RCs. **A** ^1^H NMR spectra showing time-dependent formation of benzaldehyde and 1-amino-but-3-ene (green) during the reaction of **1** (blue) with HCHO at pH 7.4. **B** Bar chart showing the percentage loss of **1**–**4** after incubation with RCs for 24 h at pH 7.4. **4** is the most promiscuous RC scavenger tested. **C** Table showing pseudo-first order rate constants for the reactions of scavengers **1**–**4** with HCHO and GA. **D** ^1^H NMR spectra, taken at different time points, of the reaction of **4** and GA at pH 7.4. Time-dependent formation of salicylaldehyde is observed (blue), while the corresponding second imine/iminium intermediate (shown as the imine, red) is observed most prominently at early time points. **E** Bar chart showing the ratio of HCHO-derived to GA-derived reaction products (combined aromatic aldehyde and second imine/iminium intermediate) after incubations of **1**, **3**, and **4** with a 1:1 mixture of HCHO and GA at pH 7.4. Scavengers **1** and **3** reacted more efficiently with HCHO (ratio > 1), while **4** reacted more efficiently with GA (ratio < 1).

We then monitored the reaction of **1** with HCHO at pH 7.4. An initial sample was prepared containing **1** (1.67 mM, diluted from a 100 mM stock solution in DMSO) and a 10-fold excess of HCHO in 100 mM sodium phosphate buffer at pH 7.4. The sample was then left to react at 298 K for 24 h before analysis by ^1^H NMR. Reaction was evidenced by the emergence of new resonances in the ^1^H NMR spectrum, which were assigned to the expected aza-Cope products benzaldehyde and 1-amino-but-3-ene. No ^1^H resonances corresponding to **1** were observed (Fig. 1A). We then conducted time-course studies. ^1^H NMR analyses during the first 24 h of reaction revealed time-dependent and HCHO-dependent formation of benzaldehyde and 1-amino-but-3-ene (Fig. 1B). However, the reaction was slow under these conditions (pseudo first order rate constant (*k*_obs_) = 8.94 × 10^−4^ s^−1^, Fig. 1C). The time-course experiments were then repeated with methylated compounds **2** and **3** in place of **1**. ^1^H NMR analysis of these samples revealed formation of benzaldehyde and either 1-amino-pent-3-ene or 1-amino-4-methyl-pent-3-ene (derived from **2** or **3**) respectively, while the reaction rates were markedly faster (Supplementary Figs. S1 and S2). The fastest to react with HCHO was di-methylated **3** (*k*_obs_ = 1.69 × 10^−3^ s^−1^, Fig. 1C and Supplementary Fig. S2); this observation supports reported time-course analyses with fluorescent aza-Cope HCHO scavengers, which revealed faster HCHO scavenging with a methylated analogue. The mono-methylated scavenger **2** reacted with HCHO at a marginally slower rate than **3** (*k*_obs_ = 1.59 × 10^−3^ s^−1^, Fig. 1C and Supplementary Fig. S1). Collectively, the time-course studies suggest the reactivity of 1-amino-but-3-enes can be modulated by altering the extent of methylation at the allylic carbon.

We then analysed the reaction rates for **1** and **3** at pH 4.5 and pH 9.5. Interestingly, the reaction was faster for all scavengers at pH 4.5 over pH 7.4 and pH 9.5 (pH 4.5: *k*_obs_ = 9.61 × 10^−4^ s^−1^ and 2.11 × 10^−3 ^s^−1^ for **1** and **3**, respectively, pH 9.5: *k*_obs_ = 4.83 × 10^−4 ^s^−1^ and 5.12 × 10^−4 ^s^−1^ for **1** and **3**, respectively). Both scavengers reacted the slowest at pH 9.5. These findings are presumably a consequence of reduced imine formation, which is classically favoured under mildly acidic conditions, and/or slower rates of the aza-Cope rearrangement, which is accelerated when the nitrogen is protonated (iminium).

Selectivity studies were then conducted. Samples containing one of **1**–**3** were prepared under our standard conditions at pH 7.4; however, this time, HCHO was replaced by one of either acetaldehyde, propionaldehyde, butyraldehyde, glyoxal, GA, glyceraldehyde, acetone, pyruvic acid, 2-keto-D-glucose or *trans*-crotonaldehyde. The samples were then monitored after 12, 24, and 48 h incubation at room temperature by ^1^H NMR. Interestingly, reactions were observed between the tested RCs and all scavengers (Fig. 1B and Supplementary Fig. S3), which indicates that 1-amino-1-phenyl-but-3-enes are not fully selective scavengers of HCHO. With **1**, no significant reaction was observed with acetaldehyde, propionaldehyde, 2-keto-D-glucose, acetone, pyruvic acid or *trans*-crotonaldehyde after 48 h (Fig. 1B). However, low-level reactions were observed with butyraldehyde and glyceraldehyde (Fig. 1B), while GA, which was not part of previous RC selectivity studies with 1-amino-but-3-enes^32–35^, was observed to react with **1** at a comparable efficiency to HCHO, reaching similar levels of conversion after 48 hours (Fig. 1B). Scavengers **2** and **3** were less selective than **1**, reacting at least moderately efficiently with acetaldehyde, propionaldehyde, butyraldehyde, glyceraldehyde, glyoxal, GA and *trans*-crotonaldehyde. Reactions of **2** with glyoxal or *trans*-crotonaldehyde were only observed at low levels after 48 h (Fig. 1B).

We then focused our studies on GA scavenging. Firstly, time-course experiments were conducted on samples containing one of **1**–**3** and GA at pH 7.4, which revealed marginally slower reactions to those observed between **1**–**3** and HCHO (*k*_obs_ = 8.17 × 10^−4 ^s^−1^, 1.07 × 10^−3 ^s^−1^ and 1.24 × 10^−3 ^s^−1^ for **1**–**3**, respectively, Fig. 1C and Supplementary Figs. S4–S6). Scavenger **3** reacted at the fastest rate, presumably due to a more efficient aza-Cope rearrangement step. To promote GA scavenging efficiency, we then synthesised and tested a new 1-amino-but-3-ene (**4**, Scheme 1)^36^. This compound contains an ortho-hydroxyl group on the phenyl ring and was designed to promote imine/iminium formation, as previously reported for phenolic benzylamines^31^. Initial time-course studies with HCHO at pH 7.4 revealed a more efficient reaction for **4**, relative to unmodified scavenger **1** (2.14 × 10^−3 ^s^−1^, Fig. 1C and Supplementary Fig. S7). We then conducted time-course experiments with **4** and GA. As expected, **4** reacted more efficiently than **1** with GA (*k*_obs_ = 3.46 × 10^−3 ^s^−1^, Fig. 1C, 1D, and Supplementary Fig. S8), which suggests that the combination of ortho-hydroxylation and di-methylation promotes reaction with GA over HCHO. In the sample with **4** and GA (and in the sample with **4** and HCHO), significant formation of a new intermediate species was also observed in the ^1^H NMR spectra, which was assigned to the imine/iminium formed after the aza-Cope rearrangement (second imine/iminium intermediate, Fig. 1D). This imine/iminium, which was not observed with scavengers **1**–**3** (at least to quantifiable levels), increased in concentration over early time-points but decreased in concentration later in the analysis period. Therefore, it appears that ortho-hydroxylation of the phenyl group induces the build-up of the second imine/iminium intermediate, which then slowly hydrolyses to the amine and salicylaldehyde products. Selectivity for GA over HCHO with **4** was further evidenced by competition experiments, where scavengers **1,** **3** and **4** were individually treated with 1:1 mixtures of HCHO and GA (each at 10-fold excess) under our standard conditions at pH 7.4. These time-course analyses revealed that **1** and **3** react preferentially with HCHO, whereas **4** reacts preferentially with GA (Fig. 1E). Further selectivity studies with **4** and our panel of RCs (24 h incubation at pH 7.4) also revealed a significant degree of promiscuity (Fig. 1B)—therefore, **4** can be considered a broad-spectrum RC scavenger.

**Fig. 2:**
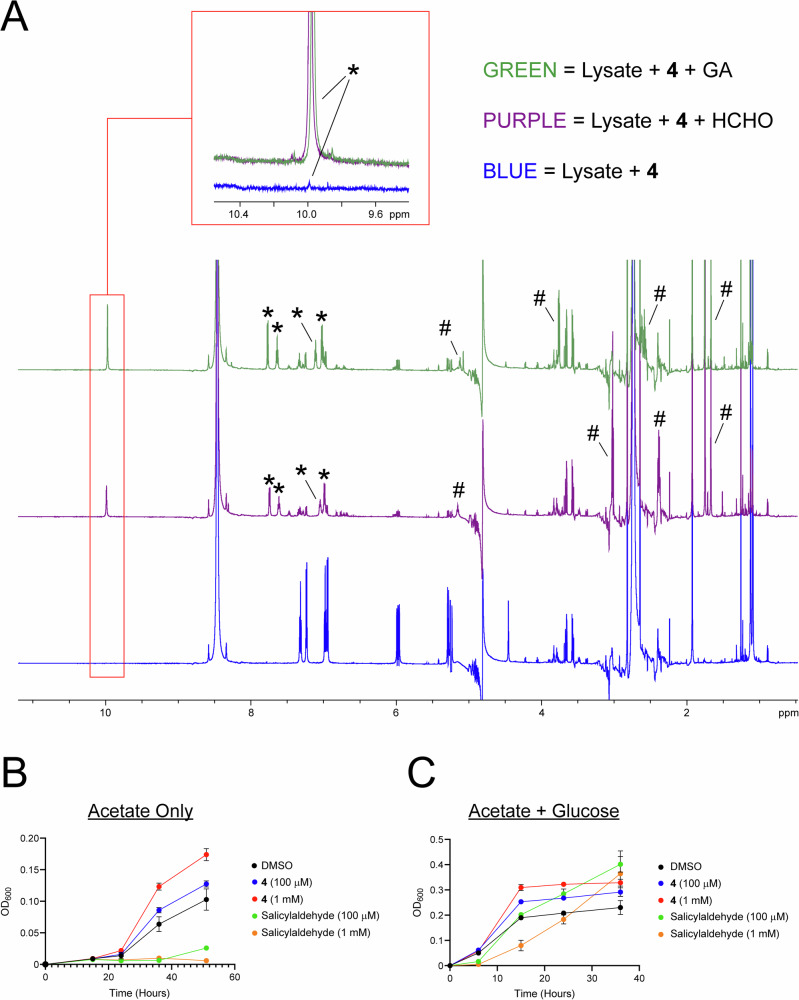
Scavenger 4 sequesters RCs in bacterial cell lysate and inhibits bacterial growth. **A** ^1^H NMR spectra showing scavenging by **4** in the cell lysate from *Escherichia coli* BL21(DE3) cell lysate after 6 h incubation at 298 K. Formation of salicylaldehyde is observed in all samples (asterisks), albeit at low levels without added HCHO or GA (blue). ^1^H resonances corresponding to the amine-containing products (1-amino-4-methyl-pent-3-ene and 2-amino-5-methyl-hex-4-enoic acid from reactions with HCHO and GA, respectively) are highlighted with hashtags. **B** Graph showing growth curves for BL21(DE3) cells grown in acetate-containing M9 minimal medium. Growth was faster in samples containing scavenger **4** or salicylaldehyde. Errors are standard deviations of the mean (*n* = 2 or 3). **C** Graph showing growth curves for BL21(DE3) cells grown in acetate- and glucose-containing M9 minimal medium. Growth was faster in the presence of glucose and was fastest in samples containing **4**. Errors are standard deviations of the mean (*n* = 3).

Having determined that **4** is an efficient HCHO and GA scavenger, we were interested in testing whether **4** can scavenge RCs in bacteria. Initially, we conducted ^1^H NMR studies on cell lysate from *Escherichia coli* BL21(DE3) cells grown in acetate-containing M9 minimal medium. Growth on this medium is proposed to invoke a reliance on the GA shunt, thus potentially leading to GA accumulation. When **4** (1 mM) was added to lysate, low-level formation of salicylaldehyde was observed after 6 h incubation at 298 K, which might suggest reaction with endogenous RCs (although some RCs might arise from the environment, Fig. 2A). Unfortunately, ^1^H resonances corresponding to amine products could not be observed unequivocally due to low intensities and signal overlap; however, addition of either HCHO or GA to the lysate (2 mM) revealed significant formation of 4-methyl-pent-3-en-1-amine and 2-amino-5-methyl-hex-4-enoic acid respectively, suggesting reaction between **4** and either HCHO or GA (Fig. 2A and Supplementary Figs. S9 and S10). We then tested whether treatment of *Escherichia coli* BL21(DE3) cells with **4** affected cell growth. Cells were grown in suspension on acetate-containing M9 minimal medium, and then treated with either **4** or salicylaldehyde immediately after inoculation (both at 100 µM and 1 mM). The optical density of each sample (600 nm) was then monitored over 51 h of incubation at 37 °C. Importantly, growth was faster after treatment with **4**, relative to DMSO-treated controls (Fig. 2B and Supplementary Data 1; *n* = 2 for 100 µM **4**; *n* = 3 for 1 mM **4**). This was in contrast to salicylaldehyde treatment, which ablated growth in the presence of acetate (at least over the first 36 h, Fig. 2B). Repeat experiments in the presence of both acetate and glucose (in triplicate, *n* = 3) revealed faster cell growth than in acetate-only medium; however, samples containing **4** still exhibited faster growth than untreated or salicylaldehyde-treated controls (Fig. 2C). Collectively, the growth inhibition and lysate experiments imply that **4** promotes the growth of BL21(DE3) cells, possibly as a consequence of aldehyde scavenging.

## Conclusions

Our studies with representative 1-amino-but-3-enes suggest they can scavenge HCHO (as previously reported) and, importantly, other RCs such as GA under biologically relevant conditions. The selectivity of the scavengers tested appears to be dependent on the sample conditions, while the reactivity and selectivity profiles were additionally dependent on the substitution pattern at the allylic carbon and the presence of a phenolic ortho-hydroxyl group.

Although the reactions between 1-amino-but-3-enes and RCs are complex, our studies enable some mechanistic hypotheses to be proposed. Firstly, it should be noted that each step of the reactions between 1-amino-but-3-enes and RCs is likely to be reversible under biologically relevant conditions (Scheme 1), which means that changes in the equilibrium positions of each step have the potential to affect the overall reaction efficiency. The first equilibrium, i.e., formation of the first imine/iminium intermediate, generally sits toward the free scavenger and RC in water; however, imine/iminium formation is more prominent with HCHO than with other aliphatic aldehydes, while the GA-derived imine/iminium is presumably stabilised by hydrogen bonding to the carboxyl group (Supplementary Scheme S1). The aza-Cope rearrangement step is likely more dynamic with HCHO due (in part) to steric factors, while for 1-amino-but-3-enes containing a 1-phenyl group (such as scavengers **1**–**4**), the second imine/iminium intermediate is more stable due to resonance, which will bias the equilibrium (Supplementary Scheme S1). The third and final equilibrium, i.e. hydrolysis of the second imine/iminium intermediate, sits to the free aldehyde and amine product in water, which drives the overall reaction forward.

Unmethylated 1-amino-but-3-enes such as **1** appear to undergo slow aza-Cope rearrangements, leading to a poor overall reaction efficiency. However, methylation at the allylic carbon promotes the aza-Cope rearrangement step, presumably via a Thorpe-Ingold-type effect (Supplementary Scheme S1). This effect should therefore lead to a more efficient scavenging reaction, as observed with scavenger **4**, while they also appear to reduce selectivity. However, this does not fully explain the observed preference of **4** for GA over HCHO. The significant build-up of the second imine/iminium intermediate with **4** (after reaction with both HCHO and GA) suggests that ortho-hydroxylation promotes its formation/stability, presumably by resonance effects and by forming an internal hydrogen bond with the nitrogen (neutral or protonated, Supplementary Scheme S1). With GA, the carboxylic acid group enables formation of an additional, presumably stabilising, hydrogen bond to the first and second imine/iminium intermediates, thus potentially promoting the reaction with GA over that with HCHO.

In conclusion, our results reveal the as-yet unrealised potential for 1-amino-but-3-enes to scavenge not only HCHO but also other biologically relevant RCs, which we hope will lead to the development of new chemical probes applicable to cellular assays, and potentially to the generation of RC-scavenging drugs. However, the toxicity of any reaction products, i.e. benzaldehyde/salicylaldehyde and amine products, as well as any off-target effects, must be considered before using 1-amino-but-3-enes in patients. Our results also emphasise the importance of conducting (bio)chemical characterisation studies, including kinetic and selectivity studies, before employing such compounds in quantitative functional and biomedical experiments.

## Methods

All methods are given in the Supplementary Information. Source data for the growth assays in BL21(DE3) cells are given in Supplementary Data 1, while ^1^H and ^13^C NMR spectra of synthesised compounds (if not otherwise included in the Main Text or Supplementary Information) are given in Supplementary Data 2. Further information on research design is available in the Nature Portfolio Reporting Summary linked to this article.

### Reporting summary

Further information on research design is available in the Nature Portfolio Reporting Summary linked to this article.

## Supplementary information

Supplementary Information

Description of Additional Supplementary Files

Supplementary Data 1

Supplementary Data 2

Reporting Summary

## Data Availability

All data are available in the Main Text, Supplementary Information, Supplementary Data, or from the corresponding author on reasonable request.
