# Supplementary material for: 1-Amino-but-3-enes scavenge formaldehyde and glyoxylic acid
Source: Commun Chem. 2026 Jan 12;9:71. doi: 10.1038/s42004-025-01873-9 (PMC12881512; doi:10.1038/s42004-025-01873-9)
Supplement: Supplementary file 1 — Supplementary Information [file 42004_2025_1873_MOESM1_ESM.pdf]

Supplementary Information

1-Amino-but-3-enes scavenge formaldehyde and glyoxylic acid

Natasha F. A. Bulman, Vicki L. Emms, Liam A. Thomas, Lilla Beja and Richard J. Hopkinson*

Leicester Institute of Structural and Chemical Biology and School of Chemistry, University of Leicester, Henry Wellcome Building, Lancaster Road, Leicester, LE1 7RH, UK. E-mail: richard.hopkinson@leicester.ac.uk

Synthesis and characterisation of scavengers

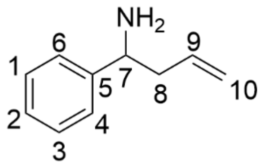

1: Benzaldehyde (250 mg; 2.36 mmol) was dissolved into 7 M NH_3 in MeOH (5 mL) for 3 hours, before allylboronic acid pinacol ester (883 µL, 4.71 mmol) was added dropwise. The reaction was then allowed to warm to room temperature and was stirred for a further 18 hours. The mixture was then acidified with 1 M $HCl_{(aq)}$ and washed with Et_2O. The aqueous was then basified using 50% w/v $NaOH_{(aq)}$ and extracted with CH_2Cl_2. The combined organic layers were washed with H_2O and brine before drying over anhydrous Na_2SO_4 and concentrated under reduced pressure. The title compound was obtained as a yellow oil (304 mg, 88%). ^1H NMR (500 MHz, $CDCl_3$, Me_4Si) δ 7.40-7.27 (4H, m, 1-CH, 3-CH, 4-CH, 6-CH), 7.24 (1H, m, 2-CH), 5.76 (1H, m, 9-CH), 5.13 (1H, app d, J = 17.0 Hz, 10-CH_2trans), 5.09 (1H, app d, J = 10.0 Hz, 10-CH_2cis), 3.97 (1H, dd, J = 8.0, 5.5 Hz, 7-CH), 2.50-2.32 (2H, m, 8-CH_2), 1.59 (2H, br s, NH_2). ^{13}C NMR (125 MHz, $CDCl_3$) δ 145.9 (5-C), 135.5 (9-C), 128.4 (1-C, 3-C), 127.0 (4-C, 6-C), 126.4 (2-C), 117.6 (10-C), 55.4 (7-C), 44.3 (8-C). MS (ES^+) m/z 132 $[M-NH_2]^+$. HRMS (ES^+) $[M-NH_2]^+$ $C_{10}H_{11}$ requires 131.0855, found 131.0861.

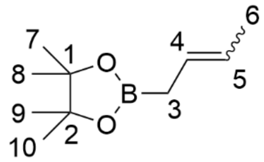

2-(But-2-en-1-yl)-4,4,5,5-tetramethyl-1,3,2-dioxaborolane: Bis(pinacolato)diboron (7.2 g, 28.3 mmol), $CsCO_3$ (0.68 g, 0.21 mmol) and MeOH (5.6 mL, 220 mmol) were added to THF (10 mL). 2-Methyl-3-buten-2-ol was then added to the mixture before heating for 6 hours under microwave irradiation (100°C). The solvent was then removed under reduced pressure and the product was purified by flash chromatography (1:9 v/v EtOAc:hexane, Rf 0.85). The product was isolated as a mixture of geometric isomers (0.66 g, 59%). ^1H NMR (major resonances only, 500 MHz, $CDCl_3$, Me_4Si) δ 5.44 (2H, m, 4-CH, 5-CH), 1.62 (5H, m, 3-CH_2, 6-CH_3), 1.23 (12H, s, 7-CH_3, 8-CH_3, 9-CH_3, 10-CH_3). ^{13}C NMR (major resonances only, 125 MHz, $CDCl_3$) δ 125.9 (5-C), 125.0 (4-C), 82.9 (1-C, 2-C), 24.7 (6-C, 7-C, 8-C, 9-C, 10-C), 18.0 (3-C). MS (ES^+) m/z 183 $[M+H]^+$.

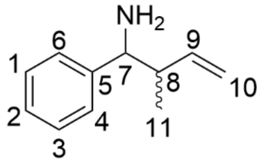

2: Benzaldehyde (77 mg, 0.73 mmol) was dissolved into 7 M NH_3 in MeOH (2 mL) under stirring for 3.5 hours. 2-(But-2-en-1-yl)-4,4,5,5-tetramethyl-1,3,2-dioxaborolane (200 mg, 1.1 mmol) was added dropwise, and the reaction was then warmed to room temperature before stirring for 24 hours. The reaction was acidified with 1 M $HCl_{(aq)}$ and washed with Et_2O before the aqueous layer was then basified using 50% w/v $NaOH_{(aq)}$ and extracted with CH_2Cl_2. The combined organic layers were then dried over anhydrous Na_2SO_4 and concentrated under reduced pressure to afford the title compound as a yellow oil (mixture of diastereomers, 69 mg, 58%). ^1H NMR (500 MHz, $CDCl_3$, Me_4Si) δ 7.28 (5H, m, 1-CH, 2-CH, 3-CH, 4-CH, 6-CH), 5.74 (1H, ddd, J = 17.0, 10.0, 8.5 Hz, 9-CH), 5.17 (1 H, m, 10-CH_2trans), 5.11 (1H, dd, J = 10.0, 2.0 Hz, 10-CH_2cis), 3.64 (1H, d, J = 8.5 Hz, 7-CH), 2.37 (1H, m, 8-CH), 1.61 (2H, br s, NH_2), 0.82 (3H, d, J = 7.5 Hz, 11-CH_3). ^{13}C NMR (125 MHz, $CDCl_3$, Me_4Si) δ 144.6 (9-C), 141.7 (5-C), 128.3 (1-C, 3-C), 127.3 (4-C, 6-C), 127.1 (2-C), 115.8 (10-C), 60.7 (7-C), 46.4 (8-C), 17.7 (11-C). MS (ES^+) m/z 162 [M+H] 145 $[M-NH_2]^+$. HRMS (ES^+) $[M-NH_2]^+$ $C_{11}H_{13}$ requires 145.1012, found 145.1017.

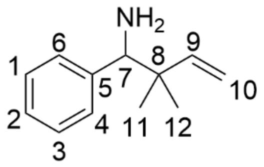

3: Benzaldehyde (250 mg; 2.36 mmol) was dissolved into 7 M NH_3 in MeOH (5 mL) and stirred for 3 hours before 3-methyl-2-butenylboronic acid pinacol ester (619 µL; 2.83 mmol) was added dropwise. The mixture was then warmed to room temperature and stirred for 18 hours. The mixture was then acidified with 1 M $HCl_{(aq)}$ and washed with Et_2O before the aqueous layer was then basified using 50% w/v $KOH_{(aq)}$ and extracted with CH_2Cl_2. The combined organic layers were washed with H_2O and brine before being dried over anhydrous Na_2SO_4 and concentrated under reduced pressure. The title compound was obtained as a yellow oil (401 mg, 97%). ^1H NMR (400 MHz, $CDCl_3$, Me_4Si) δ 7.26 (5H, m, 1-CH, 2-CH, 3-CH, 4-CH, 6-CH), 5.86 (1H, dd, J = 17.5, 10.5 Hz, 9-CH), 5.08 (1H, dd, J = 10.5, 1.5 Hz, 10-CH_2cis) 5.03 (1H, dd, J = 17.5, 1.5 Hz, 10-CH_2trans) 3.74 (1H, s, 7-CH) 1.53 (2H, br s, 2 H, NH_2) 0.98 (3H, s, 11-CH_3) 0.94 (3H, s, 12-CH_3). ^{13}C NMR (125 MHz, $CDCl_3$, Me4Si) δ 145.6 (9-C) 142.9 (5-C) 128.4 (1-C, 3-C) 127.5 (4-C, 6-C) 126.9 (2-C) 112.9 (10-C) 64.0 (7-C) 41.4 (8-C) 25.4 (11-C) 21.6 (12-C). MS

(ES^+) m/z 176 $[M+H]^+$ 159 $[M-NH_2]^+$. HRMS (ES^+) $[M+H]^+$ $C_{12}H_{18}N$ requires 176.1434, found 176.1439. $[M-NH_2]^+$ $C_{12}H_{15}$ requires 159.1168, found 159.1174.

Synthesis of **4** is previously reported.[1] Multiplet ^1H resonances are reported at the mid-point of their chemical shift ranges.

NMR Analyses

NMR experiments in buffer used 100 mM sodium phosphate buffer and were performed using either a Bruker AVIII HD NanoBay 400 MHz NMR spectrometer equipped with a 5 mm BBFO probe with z-gradients, a Bruker AVIII 500 MHz NMR spectrometer equipped with a 5 mm BBO probe with z-gradients, or a Bruker AV 500 MHz NMR spectrometer equipped with a 5 mm TXI probe with z-gradients. Experiments with bacterial cell lysate were performed using an 800 MHz Bruker AVNOE spectrometer using a 5 mm TXO cryoprobe. 3-(Trimethylsilyl)propionic-2,2,3,3-d_4 acid sodium salt (TSP) was used as an internal standard (from a stock of 10 mg mL^{-1} in D_2O) and experiments were conducted using water suppression via pre-saturation with spoil gradients (Bruker pulse program noesygppr1d). D1 = 4 seconds. All NMR experiments were conducted as single experiments (n = 1) unless otherwise stated and NMR data were processed and analysed using Topspin versions 3.6.5 or 4.4.1.

Scavenger stocks (100 mM) were prepared by dissolving solid reagents in either DMSO (for bacterial lysate studies) or DMSO-D_6. Stocks of reactive carbonyls (RCs) were prepared in 100 mM sodium phosphate buffer to a final solution of 1 M. Formaldehyde (HCHO) and glyoxylic acid (GA) stocks used for the experiments with bacterial cell lysate (1 M) were prepared in H_2O.

Selectivity experiments used a 1:10 scavenger/RC ratio. Samples were prepared in microcentrifuge tubes to a total volume of 590 µL before the scavenger (10 µL of a 100 mM stock in DMSO-D_6) was added. The samples were then transferred to 5 mm diameter NMR tubes. For samples containing **1-3**, ^1H NMR spectra were obtained after incubation at 298 K

for 12 hours, 24 hours, and 48 hours. Samples with **4** were monitored after 24 hours. Analogous control samples without RCs revealed that all scavengers are stable over 48 hours.

Samples for the rate experiments were prepared at 298 K and contained buffer, D_2O, TSP and RC (total volume = 590 µL. The scavenger (10 µL of a 100 mM stock in DMSO-D_6) was then added and the sample was immediately transferred to an NMR tube, ^1H NMR spectra were obtained consecutively for a maximum period of 1 hour after mixing. The time lapse between addition of probe and the first spectrum acquisition was measured for all experiments, while the time lapse between each spectrum acquisition was 262 seconds.

Rates were calculated by determining the increase in either benzaldehyde (for **1**-**3**) or salicylaldehyde (for **4**) concentrations in the samples over time (determined by measuring the intensity of the aldehyde ^1H resonance at δ_H 9.96 ppm relative to that of the TSP internal standard). The pseudo-first order rate constant was then obtained by applying the pseudo-first order linearised rate law:

$[A] = [A]_0 e^{-kt}$, where $[A]$ = the benzaldehyde or salicylaldehyde concentration, and t = time.

The rate constant k_{obs} was obtained from the gradient given by linear regression analysis of the plot ln[A] versus t, using GraphPad Prism 9. The R^2 value for each linear regression curve was ≥0.95.

Table S1. Values for k_{obs} and R^2 for reactions of **1-4** with HCHO and GA.

Compound	RC	pH	k_{obs}	R^2 Value
**1**	HCHO	7.4	8.94×10^{-4} s^{-1}	0.99
**1**	HCHO	4.5	9.61×10^{-4} s^{-1}	0.99
**1**	HCHO	9.5	4.83×10^{-4} s^{-1}	0.96
**1**	GA	7.4	8.17×10^{-4} s^{-1}	0.98
**2**	HCHO	7.4	1.59×10^{-3} s^{-1}	0.99
**2**	GA	7.4	1.07×10^{-3} s^{-1}	0.99
**3**	HCHO	7.4	1.69×10^{-3} s^{-1}	0.99
**3**	HCHO	4.5	2.11×10^{-3} s^{-1}	0.99
**3**	HCHO	9.5	5.12×10^{-4} s^{-1}	0.95
**3**	GA	7.4	1.24×10^{-3} s^{-1}	0.97
**4**	HCHO	7.4	2.14×10^{-3} s^{-1}	0.99
**4**	GA	7.4	3.46×10^{-3} s^{-1}	0.98

Studies on bacterial cell lysate

Escherichia coli BL21(DE3) cells were grown in pre-prepared M9 minimal medium containing 28 mM sodium acetate (30 mL of medium). After growing at 37°C with shaking for 18 hours, the cells were pelleted by centrifugation (4500 rpm for 10 minutes) before removal of the medium and resuspension in 3 mL of 50 mM ammonium formate buffer in H_2O, pH 7.5. Cell lysis was achieved by subjecting the resuspended pellet to sonication on ice at 8-10 microns amplitude for 30 seconds (2 seconds ON, 2 seconds OFF). After centrifugation (4500 rpm for 10 minutes), the lysate stock, which was used for all the NMR experiments, was collected and kept on ice. ^1H NMR experiments were then conducted immediately by preparing samples containing aliquots of the lysate stock (512 µL), 4 (6 µL of a 100 mM stock in DMSO), TSP (10 µL of a 10 mg mL^{-1} stock in D_2O), D_2O (60 µL), and either H_2O, HCHO or GA (total volume = 600 µL). The total HCHO/GA concentration was 2 mM. After preparation in microcentrifuge tubes, the samples were immediately transferred to NMR tubes and incubated at 298 K before ^1H NMR analysis.

Bacterial growth studies

Escherichia coli BL21(DE3) cells were grown in pre-prepared M9 minimal medium containing 28 mM sodium acetate (30 mL of medium), as described above. 50 µL of the medium was then added to fresh medium containing either DMSO, **4** (from a stock in DMSO) or salicylaldehyde (from a stock in DMSO). Total sample volumes were 1 mL. All experiments were conducted in triplicate (distinct samples) apart from timepoints beyond 15 hours with 100 µM **4** or 100 µM salicylaldehyde, which were duplicates. Triplicate samples were prepared containing an additional 2 µL of 20% wt. glucose in H_2O. Cell growth was monitored by measuring the optical density (600 nm) of the samples over time.

Figures

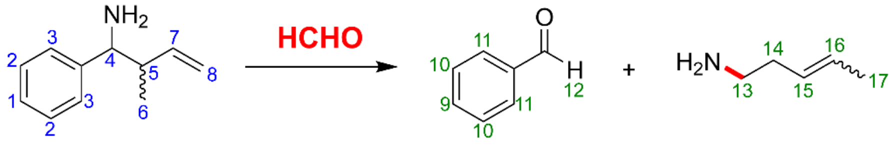

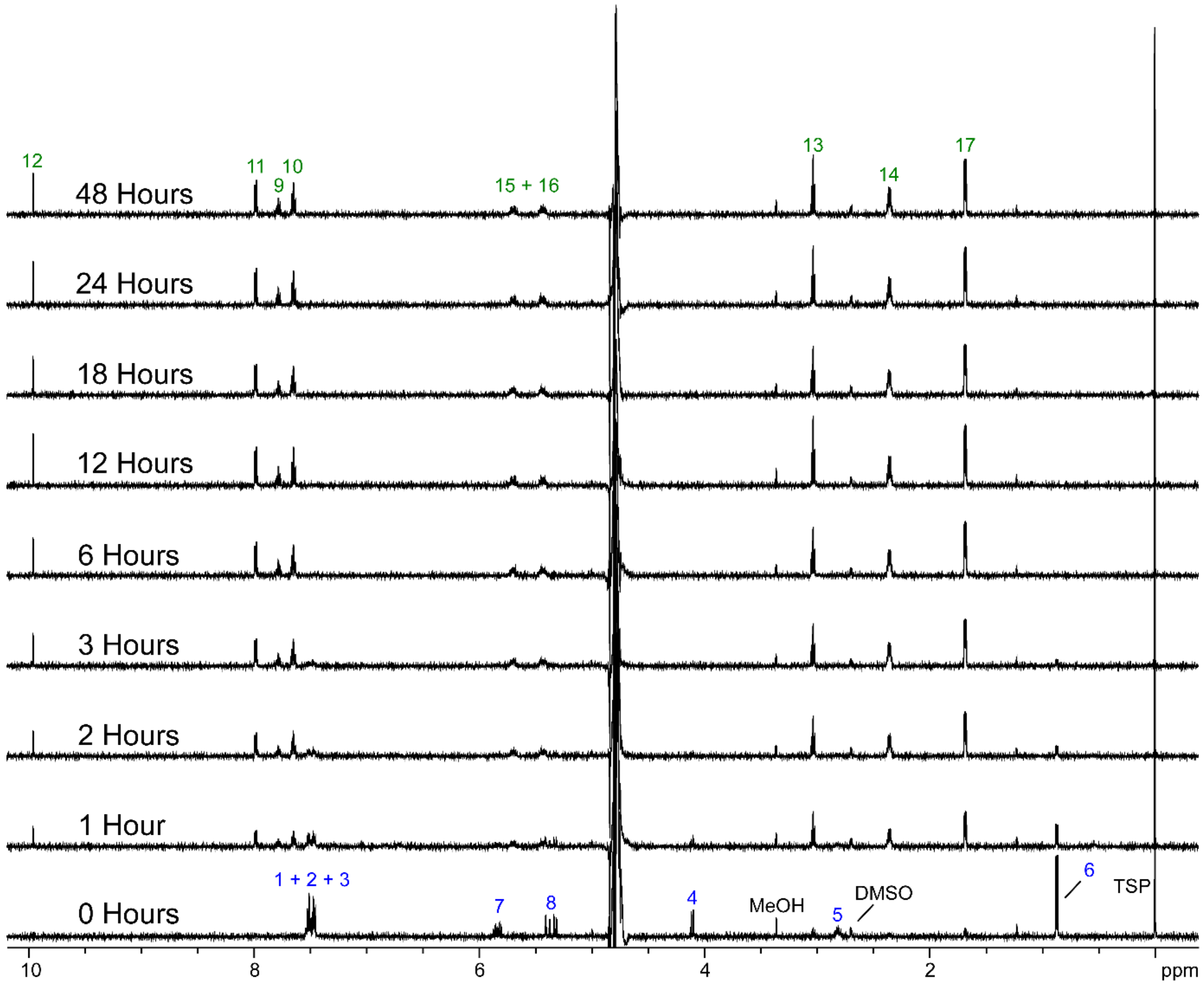

Figure S1. ^{1}H NMR spectra showing time-dependent formation of benzaldehyde and 1-amino-pent-3-ene (green) during the reaction of **2** (blue) with HCHO at pH 7.4.

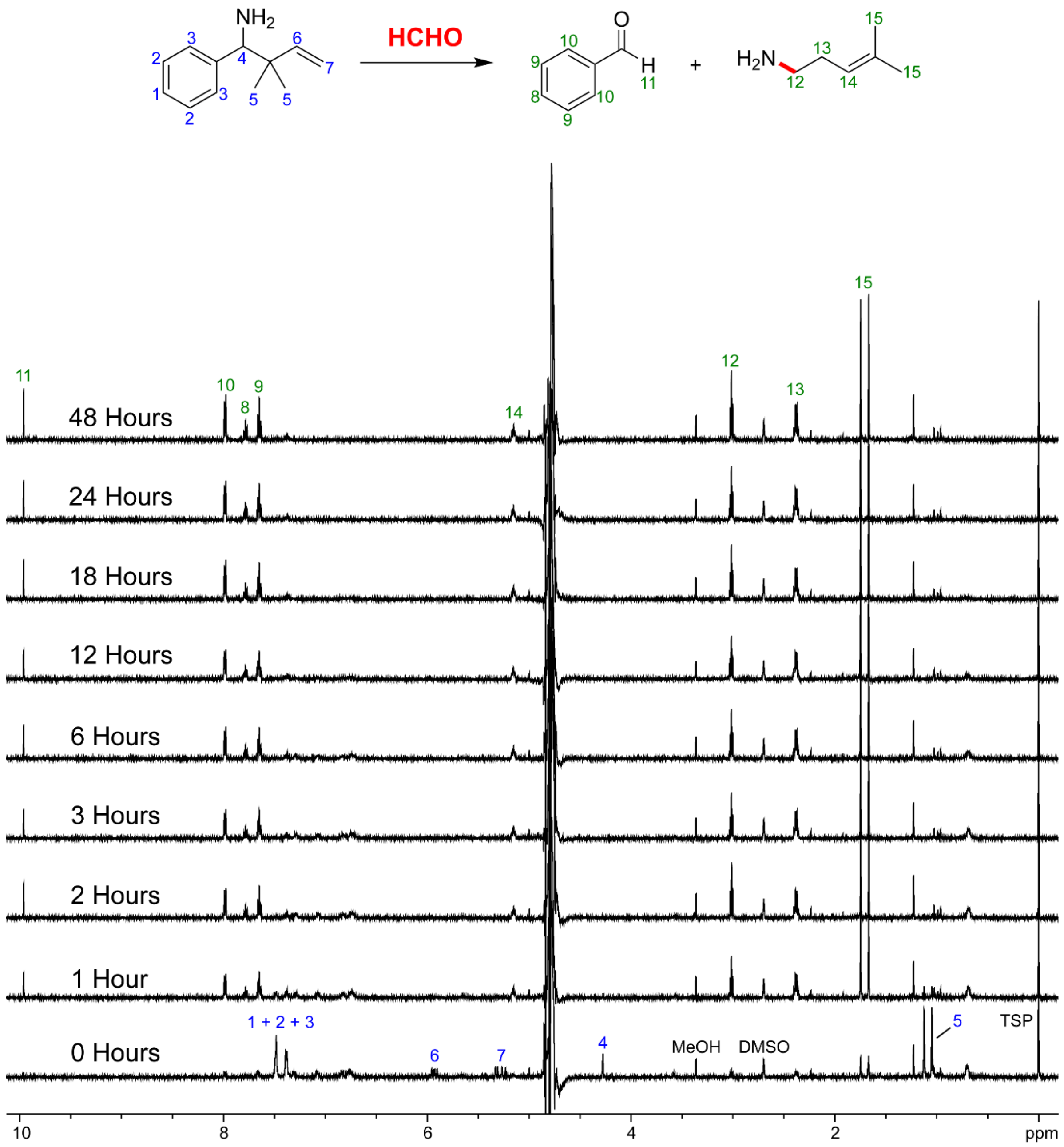

Figure S2. ^{1}H NMR spectra showing time-dependent formation of benzaldehyde and 1-amino-4-methyl-pent-3-ene (green) during the reaction of **3** (blue) with HCHO at pH 7.4.

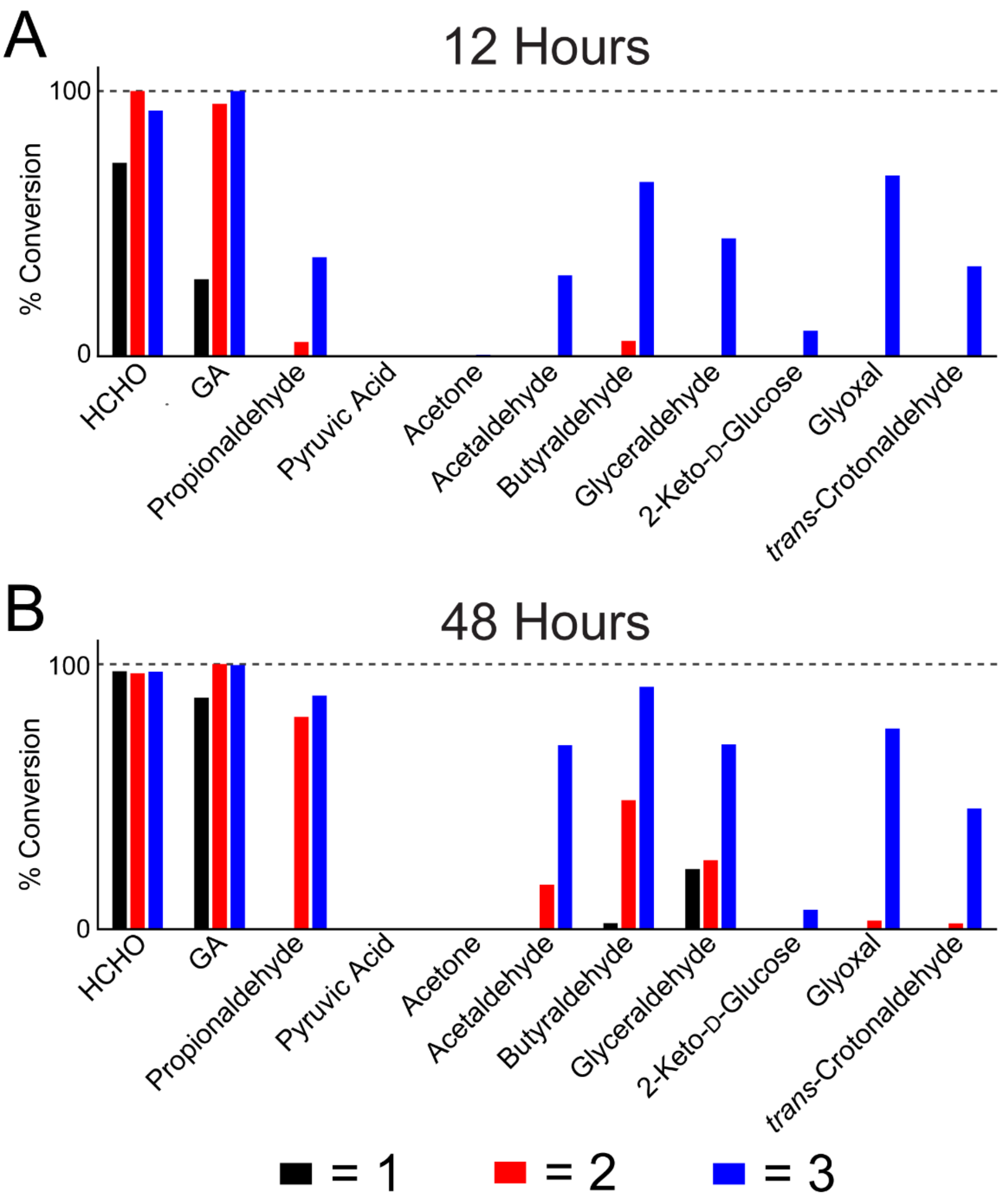

Figure S3. Bar charts showing the percentage conversion of **1**-**3** to benzaldehyde after incubation with RCs for 12 hours (A) and 48 hours (B) at pH 7.4.

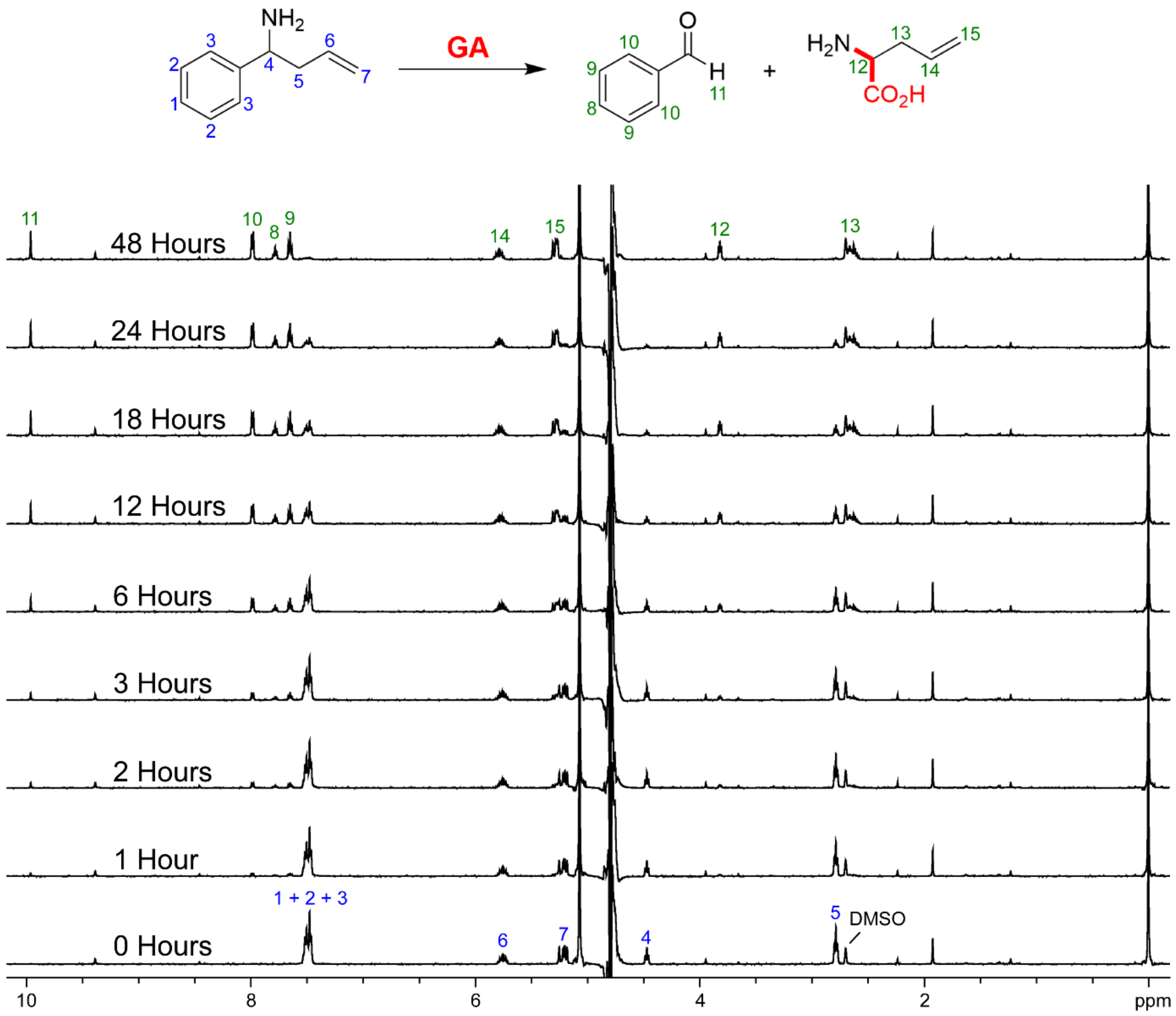

Figure S4. ^{1}H NMR spectra showing time-dependent formation of benzaldehyde and 1-amino-1-carboxy-butene (green) during the reaction of **1** (blue) with GA at pH 7.4.

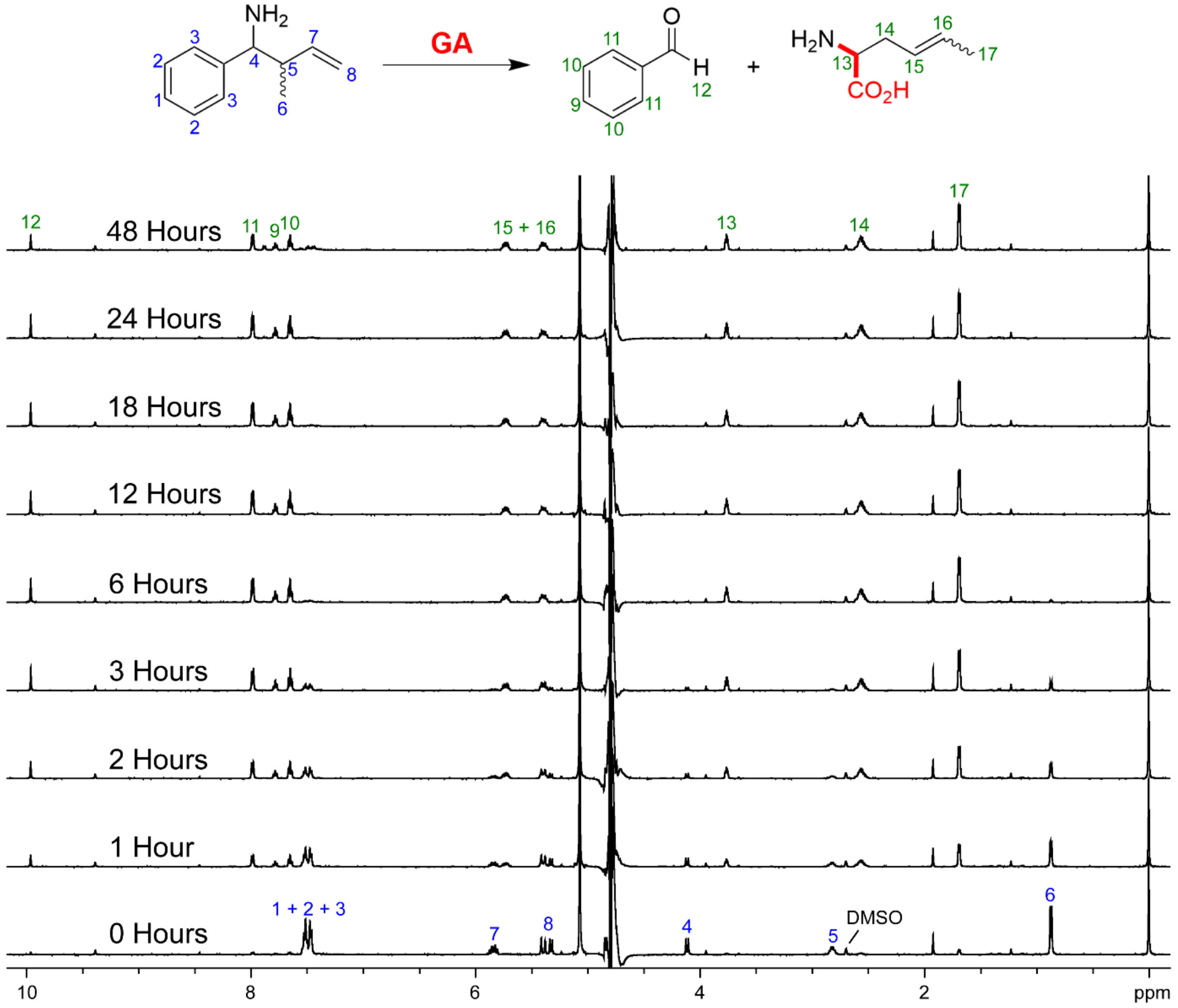

Figure S5. ^{1}H NMR spectra showing time-dependent formation of benzaldehyde and 1-amino-1-carboxy-pent-3-ene (green) during the reaction of **2** (blue) with GA at pH 7.4.

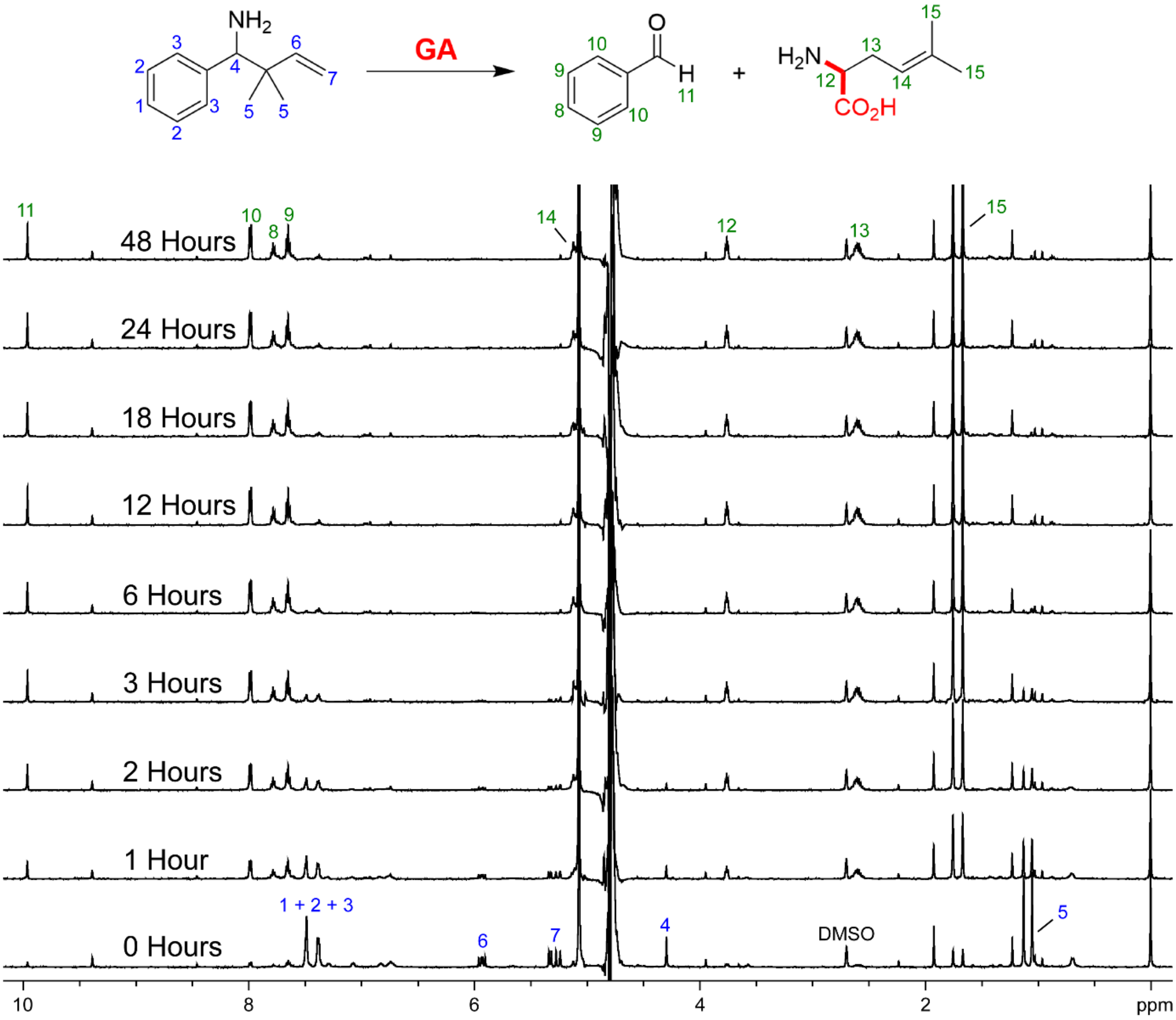

Figure S6. ^{1}H NMR spectra showing time-dependent formation of benzaldehyde and 1-amino-1-carboxy-4-methyl-pent-3-ene (green) during the reaction of **3** (blue) with GA at pH 7.4.

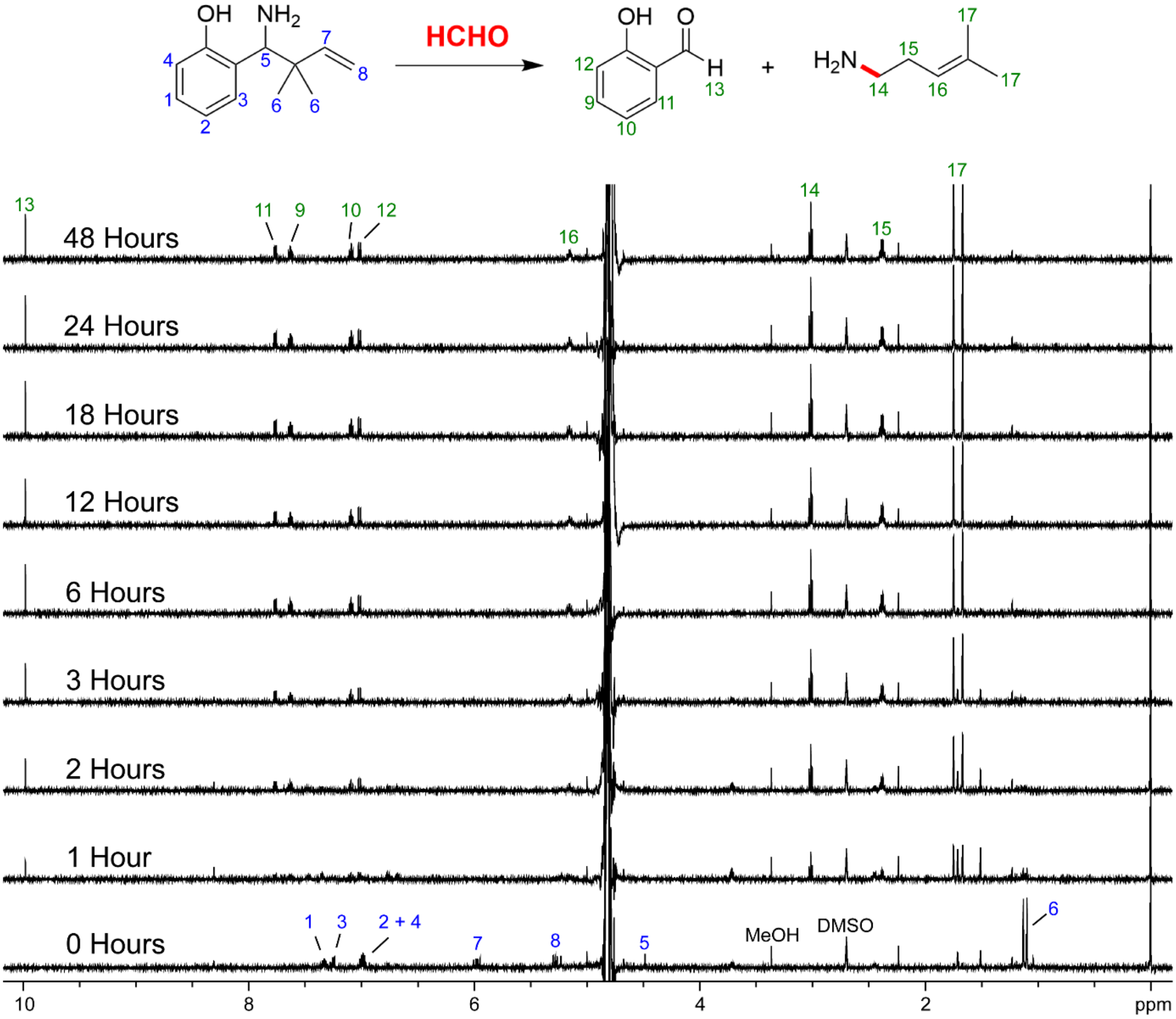

Figure S7. ^{1}H NMR spectra showing time-dependent formation of salicylaldehyde and 1-amino-4-methyl-pent-3-ene (green) during the reaction of **4** (blue) with HCHO at pH 7.4.

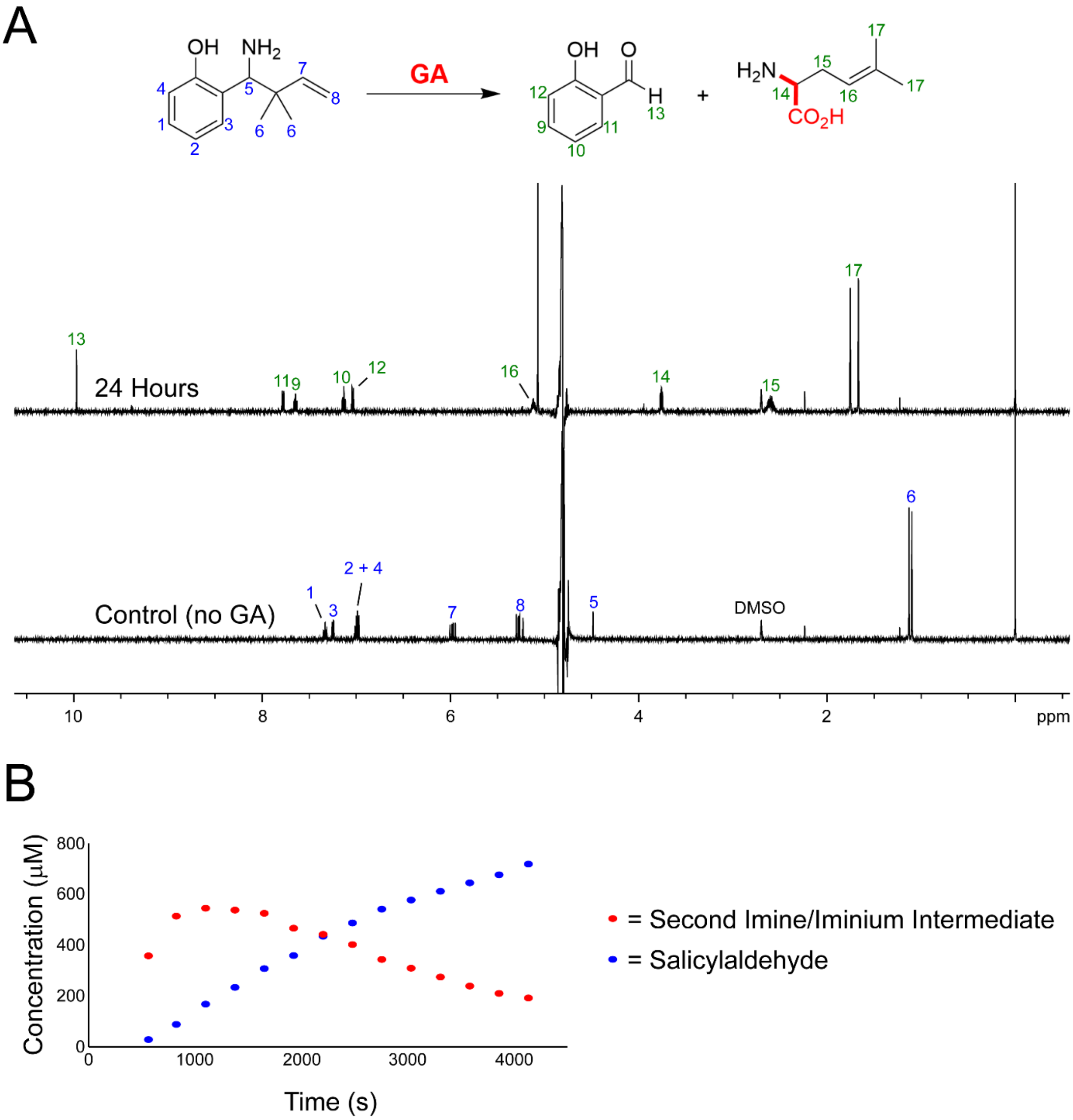

Figure S8. (A) ^{1}H NMR spectra showing time-dependent formation of salicylaldehyde and 2-amino-5-methyl-hex-4-enoic acid (green) during the reaction of **4** (blue) with GA at pH 7.4. (B) Graph showing time-dependent formation of salicylaldehyde (blue) and the second imine/iminium intermediate (red) during the reaction of **4** with GA at pH 7.4.

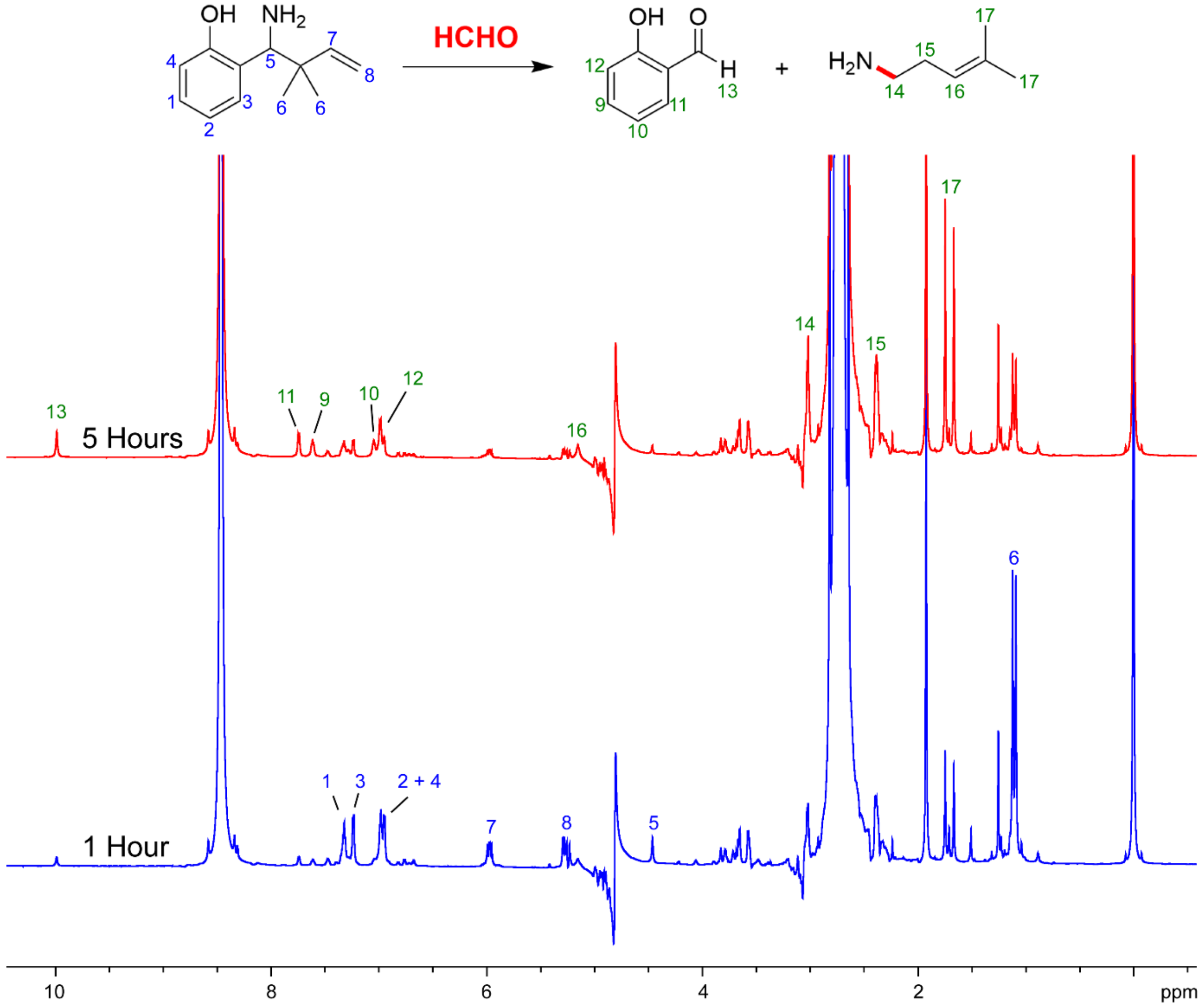

Figure S9. ^{1}H NMR spectra showing scavenging of HCHO by **4** in cell lysate from *Escherichia coli* BL21(DE3) cell lysate after one hour (blue) and 5 hours (red).

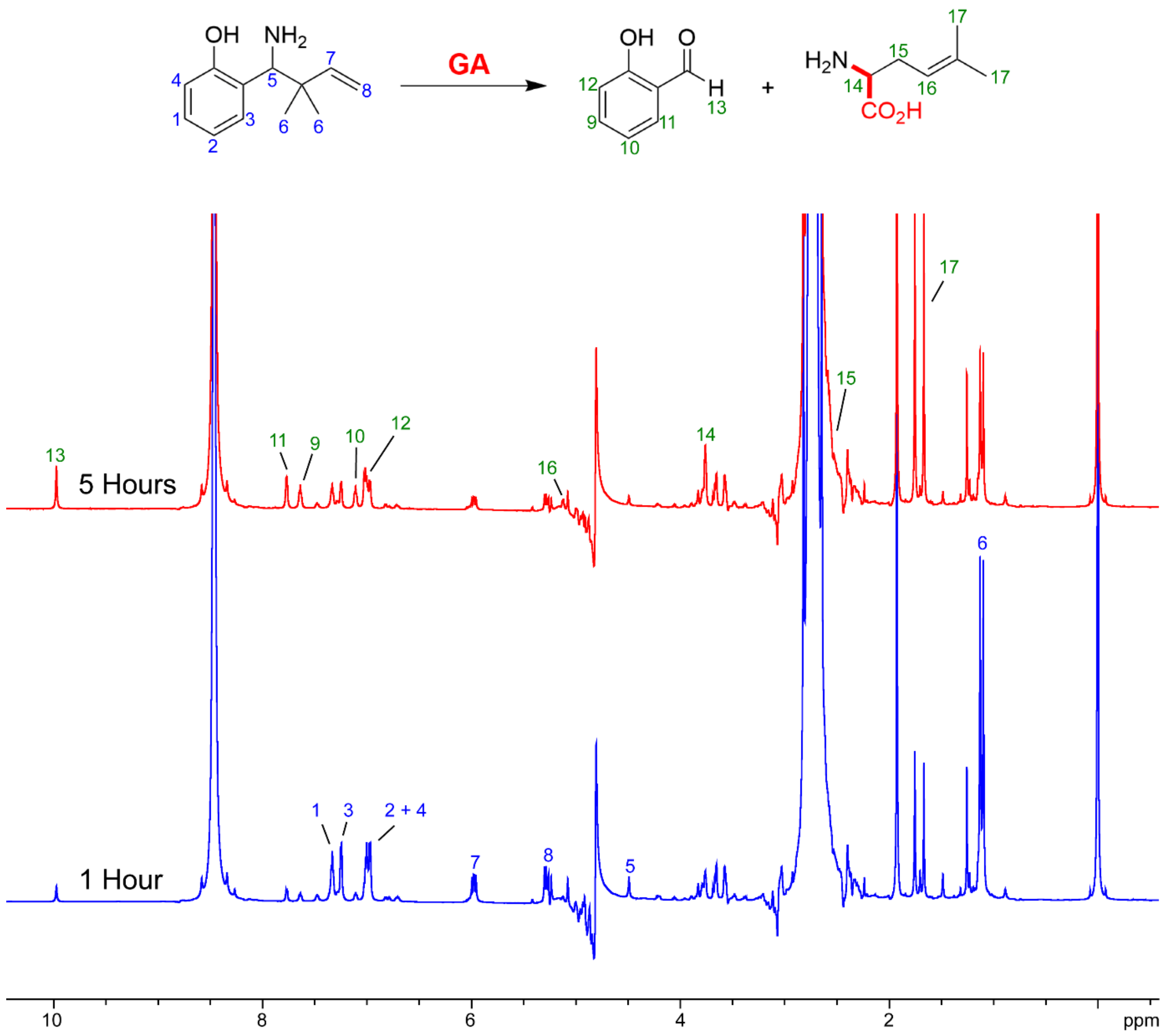

Figure S10. ^{1}H NMR spectra showing scavenging of GA by **4** in cell lysate from *Escherichia coli* BL21(DE3) cell lysate after one hour (blue) and 5 hours (red).

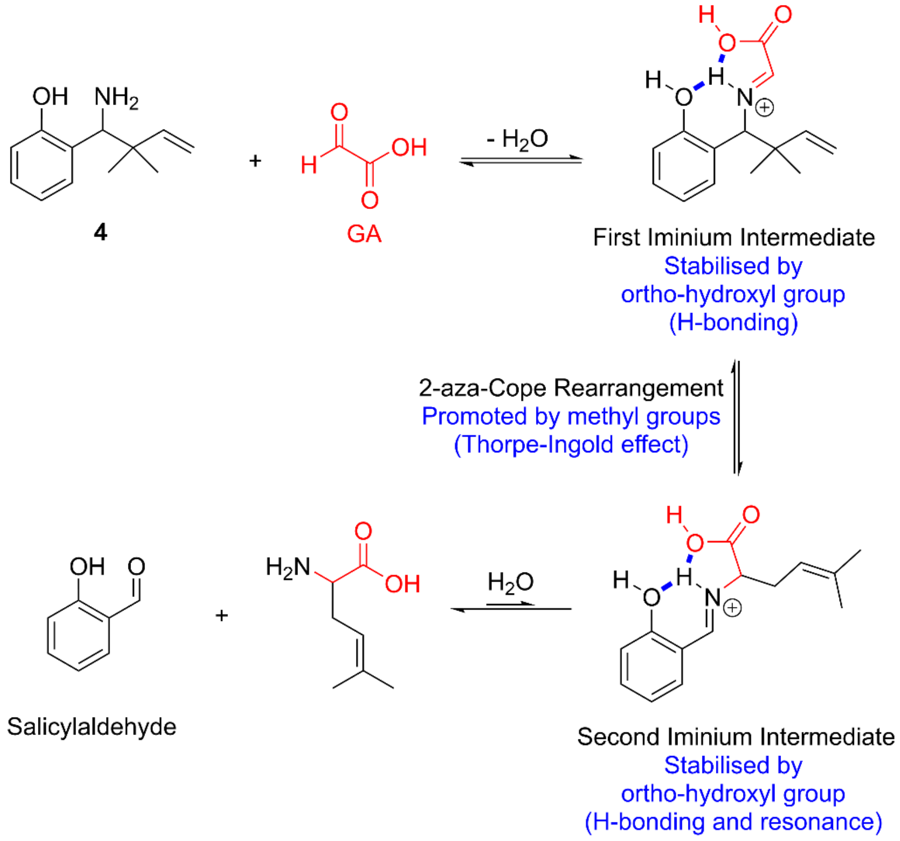

Scheme S1. Scheme showing the scavenging of GA by **4**. The first equilibrium, i.e. formation of the first imine/iminium intermediate (shown here as the iminium) is promoted by the ortho-hydroxyl group of **4** due to hydrogen bonding (H-bonding) between the hydroxyl and imino groups (blue bond). The second equilibrium, i.e. the 2-aza-Cope rearrangement step, is promoted by the two methyl groups via a Thorpe-Ingold effect, while the second imine/iminium intermediate is stabilised by H-bonding involving the ortho-hydroxyl group, and by resonance that is promoted by the electron-rich phenol. The third equilibrium favours imine/iminium hydrolysis in water. The reaction with GA is further promoted by additional H-bonding between the imino group and the carboxylic acid group (blue bonds).

Supplementary Reference

1 Chothia, S. Y. et al. Formaldehyde quantification using gas chromatography–mass spectrometry reveals high background environmental formaldehyde levels. Sci Rep. 14, 20621 (2024).
