# Supplementary material for: 1-Amino-but-3-enes scavenge formaldehyde and glyoxylic acid
Source: Commun Chem. 2026 Jan 12;9:71. doi: 10.1038/s42004-025-01873-9 (PMC12881512; doi:10.1038/s42004-025-01873-9)
Supplement: Supplementary file 2 — Description of Additional Supplementary Files [file 42004_2025_1873_MOESM2_ESM.pdf]

Description of Additional Supplementary Files:

File: Supplementary Data 1

Description: Source data for the bacterial growth assays with BL21(DE3) cells (Figure 2B/C).

File: Supplementary Data 2

Description: ^{1}H and ^{13}C NMR spectra of synthesised compounds (if not otherwise included in the Main Text or Supplementary Information).
