# Supplementary material for: 1-Amino-but-3-enes scavenge formaldehyde and glyoxylic acid
Source: Commun Chem. 2026 Jan 12;9:71. doi: 10.1038/s42004-025-01873-9 (PMC12881512; doi:10.1038/s42004-025-01873-9)

$^{13}C$ NMR spectrum ($^{1}H$-decoupled) of **1**:

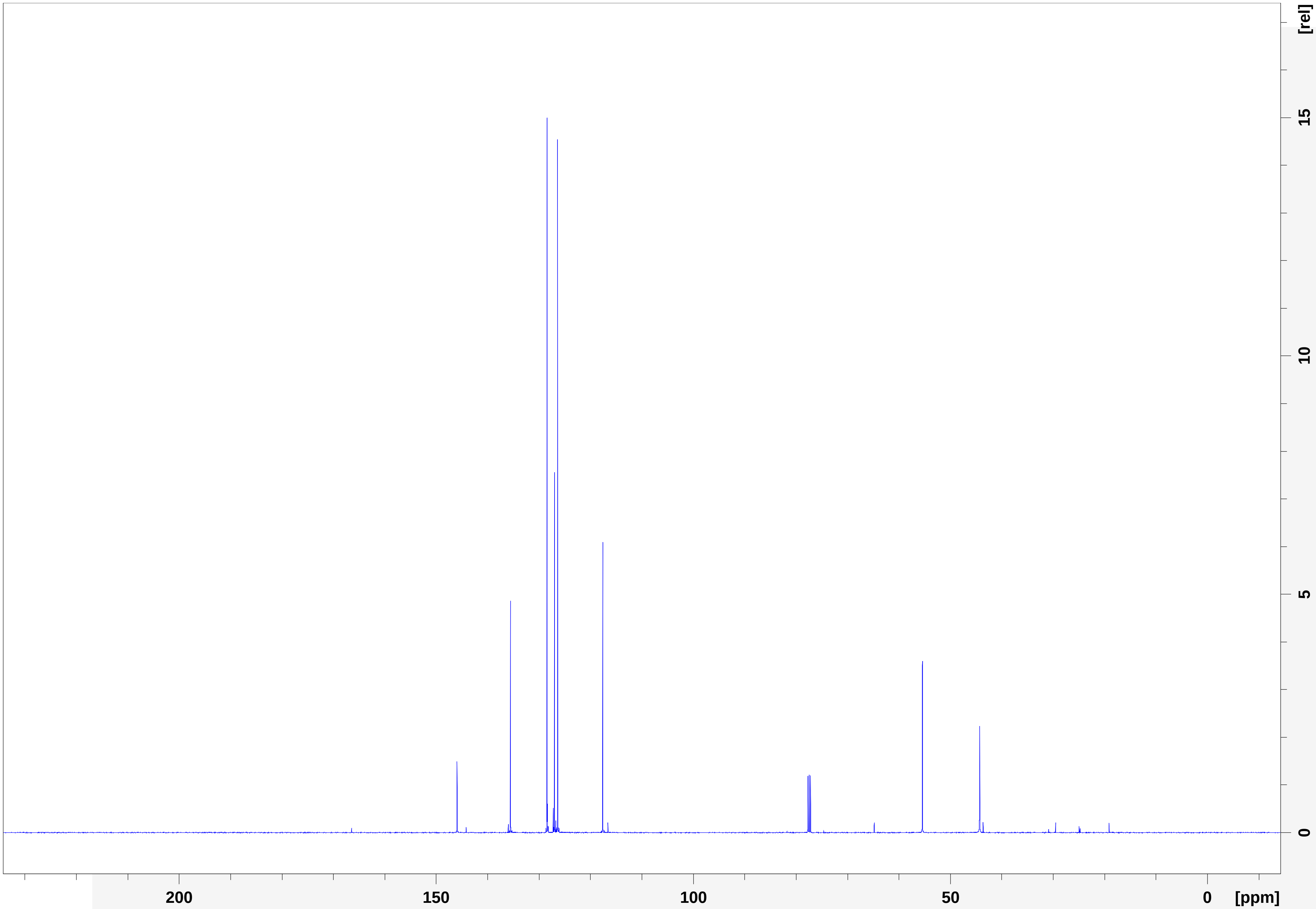

$^1H$ NMR spectrum of 2-(but-2-en-1-yl)-4,4,5,5-tetramethyl-1,3,2-dioxaborolane:

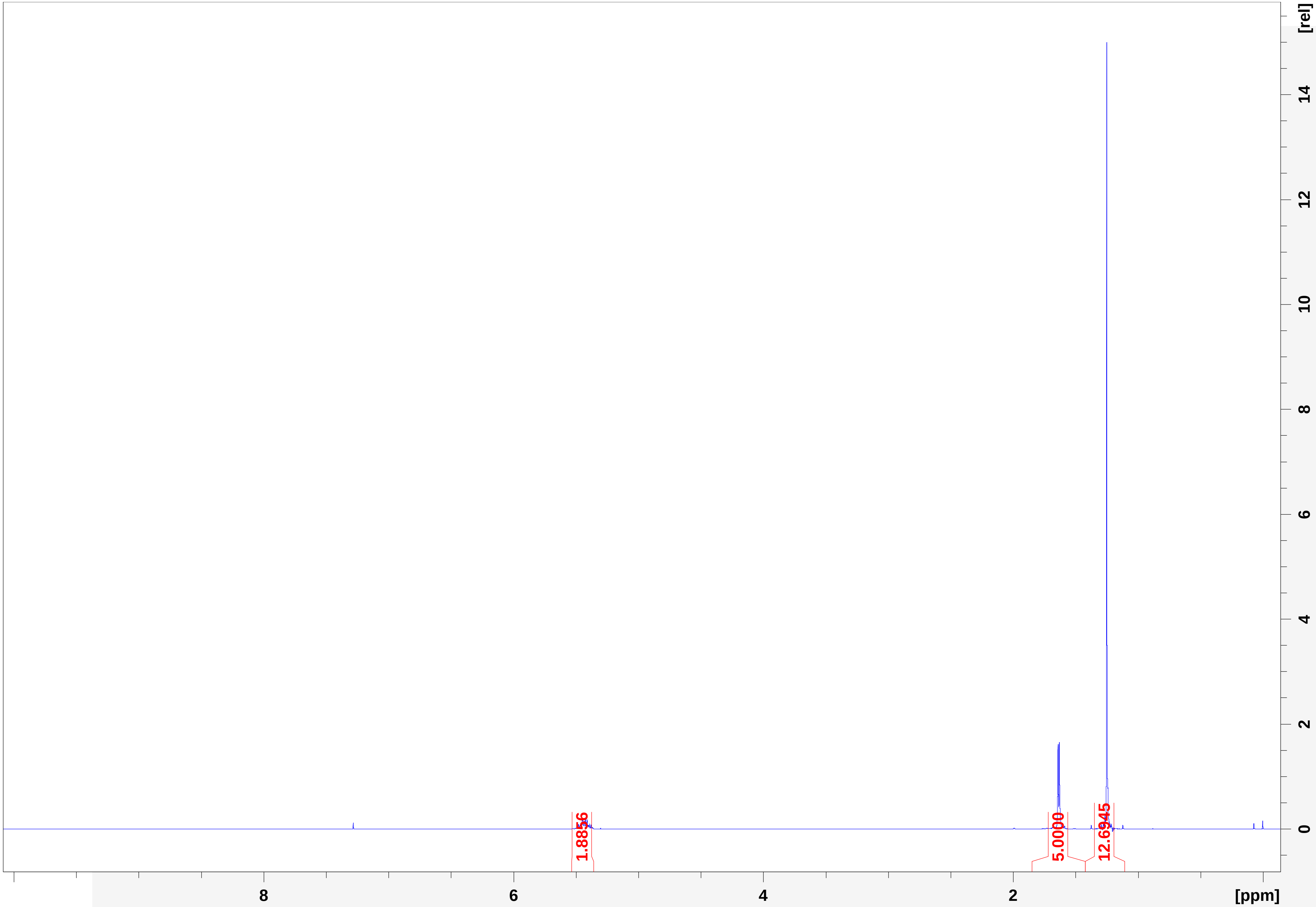

$^{13}C$ NMR spectrum ($^{1}H$-decoupled) of 2-(but-2-en-1-yl)-4,4,5,5-tetramethyl-1,3,2-dioxaborolane:

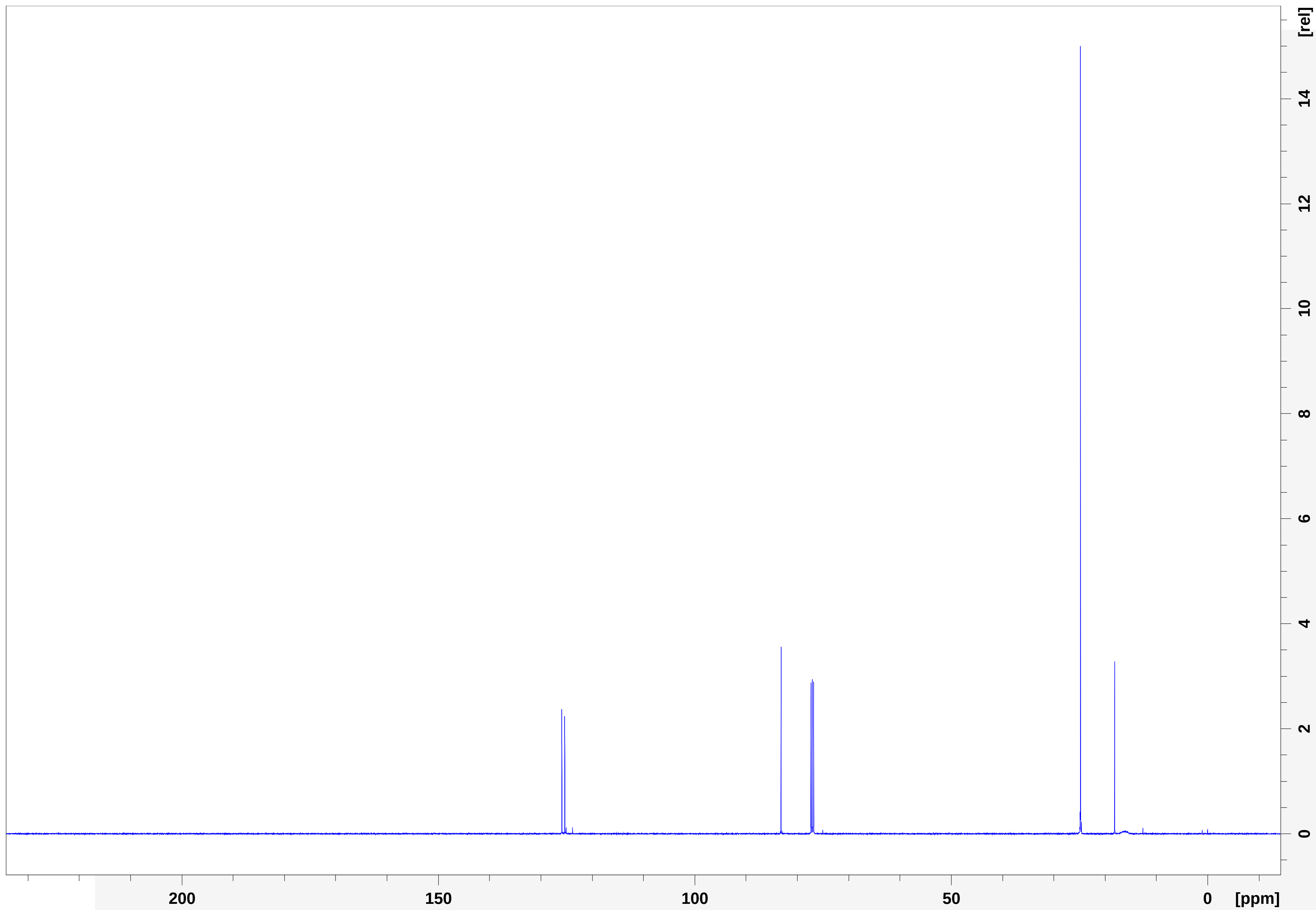

$^{13}$C NMR spectrum ($^{1}$H-decoupled) of **2**:

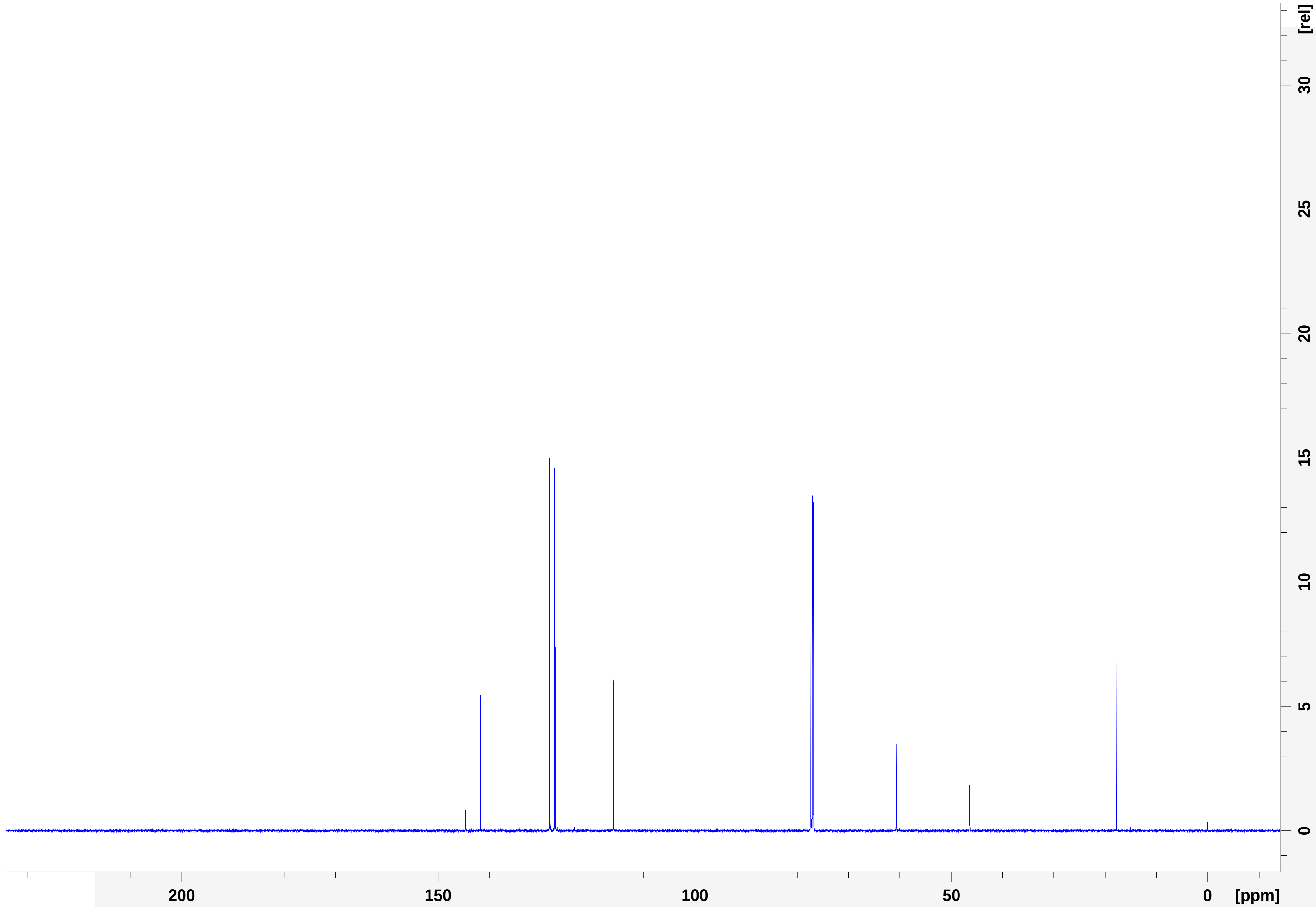

$^{13}$C NMR spectrum ($^{1}$H-decoupled) of **3**:

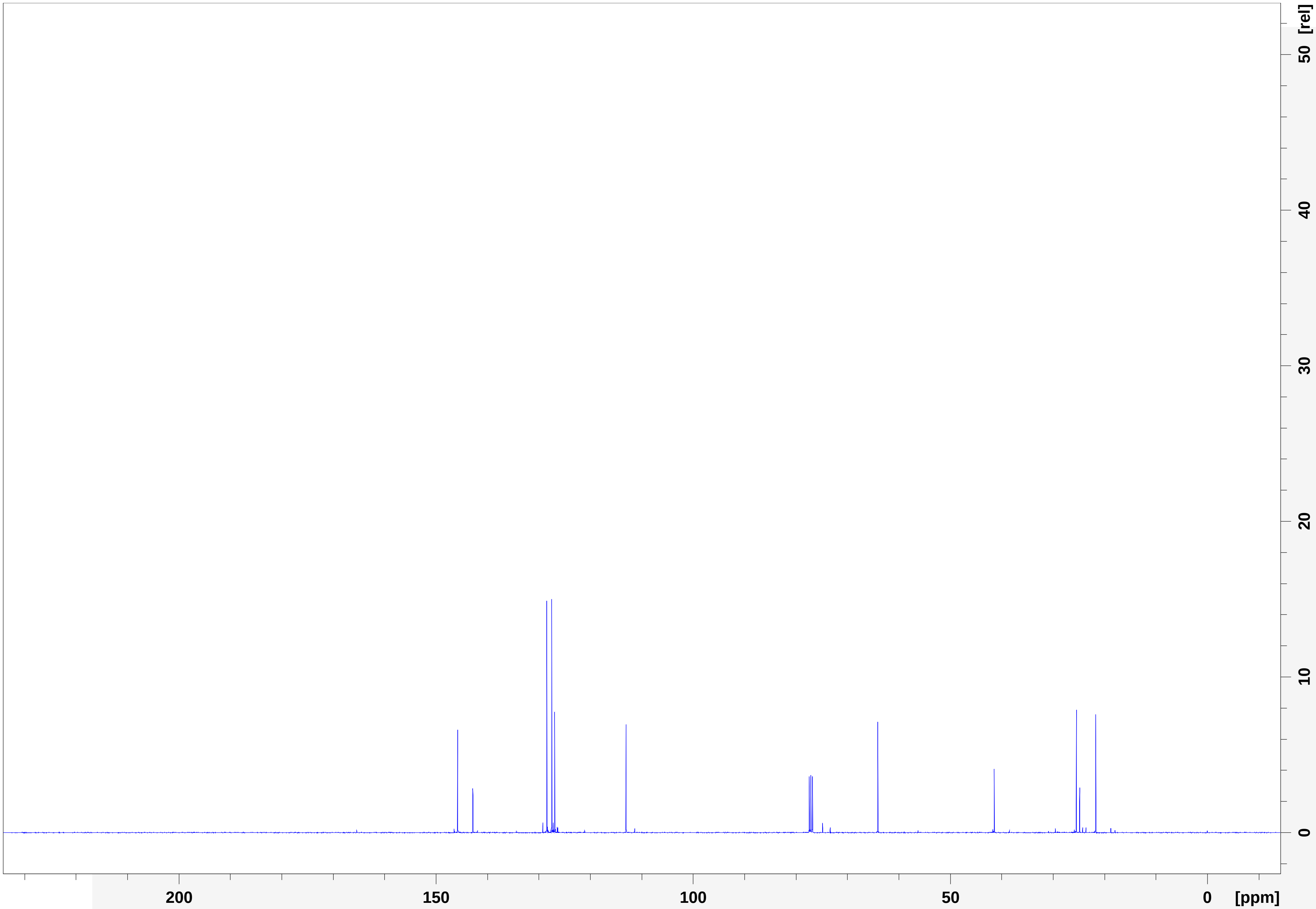

$^{13}C$ NMR spectrum ($^{1}H$-decoupled) of **4**:

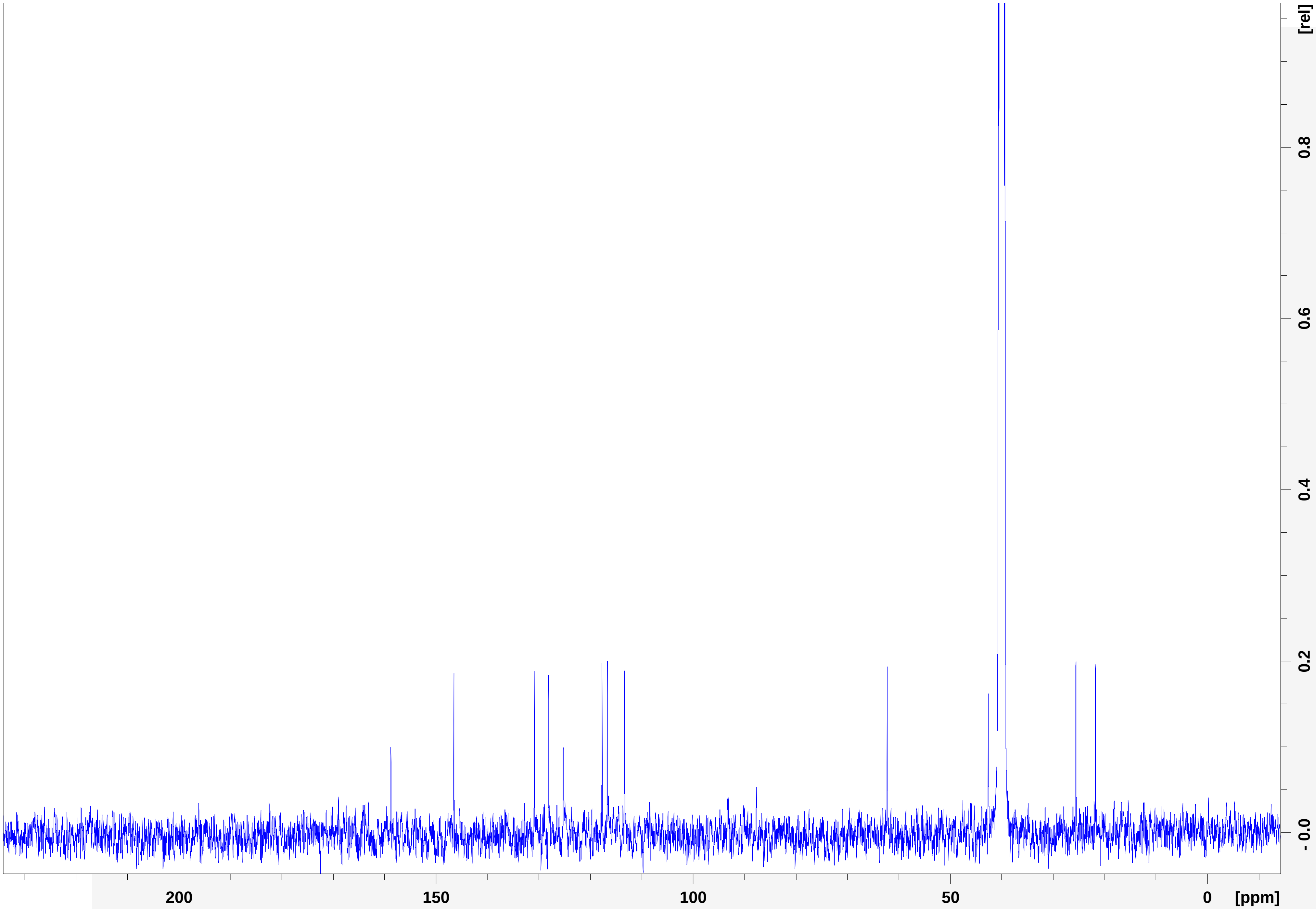

Supplement: Supplementary file 4 — Supplementary Data 2 [file 42004_2025_1873_MOESM4_ESM.pdf]
